# A pedestrian approach to the invariant Gibbs measures for the 2-*d* defocusing nonlinear Schrödinger equations

**DOI:** 10.1007/s40072-018-0112-2

**Published:** 2018-03-26

**Authors:** Tadahiro Oh, Laurent Thomann

**Affiliations:** 1 School of Mathematics, The University of Edinburgh and The Maxwell Institute for the Mathematical Sciences, James Clerk Maxwell Building, The King’s Buildings, Peter Guthrie Tait Road, Edinburgh, EH9 3FD UK; 2 Institut Élie Cartan, Université de Lorraine, B.P. 70239, 54506 Vandoeuvre-lès-Nancy Cedex, France

**Keywords:** Nonlinear Schrödinger equation, Gibbs measure, Wick ordering, Hermite polynomial, Laguerre polynomial, White noise functional, 35Q55

## Abstract

We consider the defocusing nonlinear Schrödinger equations on the two-dimensional compact Riemannian manifold without boundary or a bounded domain in $\mathbb {R}^2$. Our aim is to give a pedagogic and self-contained presentation on the Wick renormalization in terms of the Hermite polynomials and the Laguerre polynomials and construct the Gibbs measures corresponding to the Wick ordered Hamiltonian. Then, we construct global-in-time solutions with initial data distributed according to the Gibbs measure and show that the law of the random solutions, at any time, is again given by the Gibbs measure.

## Introduction

### Nonlinear Schrödinger equations

Let $(\mathcal {M},g)$ be a two-dimensional compact Riemannian manifold without boundary or a bounded domain in $\mathbb {R}^2$. We consider the defocusing nonlinear Schrödinger equation (NLS):

1.1 $$\begin{aligned} \left\{ \begin{array}{l} i \partial _tu + \Delta _g u = |u|^{k-2}u \\ u|_{t = 0} = \phi , \end{array}\right. \qquad (t,x) \in \mathbb {R}\times \mathcal {M}, \end{aligned}$$

where $\Delta _g$ stands for the Laplace–Beltrami operator on $\mathcal {M}$, $k = 2m \ge 4$ is an even integer, and the unknown is the function $u: \mathbb {R}\times \mathcal {M}\longrightarrow \mathbb {C}$.

The aim of this article is to give a pedagogic and self-contained^1^ presentation on the construction of an invariant Gibbs measure for a renormalized version of (1.1). In particular, we present an elementary Fourier analytic approach to the problem in the hope that this will be accessible to readers (in particular those in dispersive PDEs) without prior knowledge in quantum field theory and/or stochastic analysis. In order to make the presentation simpler, we first detail the case of the flat torus $\mathcal {M}=\mathbb {T}^{2}$, where $\mathbb {T}= \mathbb {R}/(2\pi \mathbb {Z})$. Namely, we consider

1.2 $$\begin{aligned} \left\{ \begin{array}{l} i \partial _tu + \Delta u = |u|^{k-2}u \\ u|_{t = 0} = \phi , \end{array}\right. \qquad (t,x) \in \mathbb {R}\times \mathbb {T}^2. \end{aligned}$$

The Eq. (1.2) is known to possess the following Hamiltonian structure:

1.3 $$\begin{aligned} \partial _tu = -\, i \frac{\partial H}{\partial \overline{u}}, \end{aligned}$$

where $H = H(u)$ is the Hamiltonian given by

1.4 $$\begin{aligned} H(u) = \frac{1}{2}\int _{\mathbb {T}^2} |\nabla u|^2dx + \frac{1}{k} \int _{\mathbb {T}^2} |u|^k dx. \end{aligned}$$

Moreover, the mass

$$\begin{aligned} M(u) = \int _{\mathbb {T}^2} |u|^2 dx \end{aligned}$$

is also conserved under the dynamics of (1.2).

### Gibbs measures

Given a Hamiltonian flow on $\mathbb {R}^{2n}$:

1.5 $$\begin{aligned} \left\{ \begin{array}{l} \dot{p}_j = \frac{\partial H}{\partial q_j} \\ \dot{q}_j = - \frac{\partial H}{\partial p_j} \end{array}\right. \end{aligned}$$

with Hamiltonian $H (p, q)= H(p_1, \ldots , p_n, q_1, \ldots , q_n)$, Liouville’s theorem states that the Lebesgue measure $\prod _{j = 1}^n dp_j dq_j$ on $\mathbb {R}^{2n}$ is invariant under the flow. Then, it follows from the conservation of the Hamiltonian *H* that the Gibbs measures $e^{-\beta H(p, q)} \prod _{j = 1}^{n} dp_j dq_j$ are invariant under the dynamics of (1.5). Here, $\beta > 0$ denotes the reciprocal temperature.

NLS (1.2) is a Hamiltonian PDE, where the Hamiltonian is conserved under its dynamics. Thus by drawing an analogy to the finite dimensional setting, one expects the Gibbs measure of the form^2^:

1.6 $$\begin{aligned} \text {``}dP^{(2m)}_2= Z^{-1} \exp (- \beta H(u))du\text {''} \end{aligned}$$

to be invariant under the dynamics of (1.2).^3^ As it is, (1.6) is merely a formal expression and we need to give a precise meaning. From (1.4), we can write (1.6) as

1.7 $$\begin{aligned} \text {``}dP^{(2m)}_2= Z^{-1} e^{-\frac{1}{2m} \int |u|^{2m} dx} e^{-\frac{1}{2} \int |\nabla u |^2 dx} du\text {''}. \end{aligned}$$

This motivates us to define the Gibbs measure $P^{(2m)}_2$ as an absolutely continuous (probability) measure with respect to the following massless Gaussian free field: $d\mu = \widetilde{Z}^{-1} \exp \big (-\frac{1}{2} \int |\nabla u |^2 dx\big ) du.$ In order to avoid the problem at the zeroth frequency, we instead consider the following massive Gaussian free field:

1.8 $$\begin{aligned} d\mu = \widetilde{Z}^{-1} e^{-\frac{1}{2} \int |\nabla u |^2 dx - \frac{1}{2}\int |u|^2 dx } du \end{aligned}$$

in the following. Note that this additional factor replaces $-H(u)$ by $-H(u) - \frac{1}{2} M(u)$ in the formal definition (1.6) of $P^{(2m)}_2$. In view of the conservation of mass, however, we still expect $P^{(2m)}_2$ to be invariant if we can give a proper meaning to $P^{(2m)}_2$.

It is well known that $$\mu $$ in (1.8) corresponds to a mean-zero Gaussian free field on $$\mathbb {T}^2$$. More precisely, $$\mu $$ is the mean-zero Gaussian measure on $$H^s(\mathbb {T}^2)$$ for any $$ s< 0$$ with the covariance operator $$Q_s = (\text {Id}-\Delta )^{-1+s}$$. Recall that a covariance operator *Q* of a mean-zero probability measure $$\mu $$ on a Hilbert space $$\mathcal {H}$$ is a trace class operator, satisfying

1.9 $$\begin{aligned} \int _{\mathcal {H}} \langle f, u \rangle _\mathcal {H}\overline{\langle h, u \rangle }_\mathcal {H}d\mu (u) = \langle Q f, h \rangle _\mathcal {H} \end{aligned}$$

for all $$f, h \in \mathcal {H}$$.

We can also view the Gaussian measure $\mu$ as the induced probability measure under the map^4^:

1.10 $$\begin{aligned} \omega \in \Omega \longmapsto u(x) = u(x; \omega ) = \sum _{n \in \mathbb {Z}^2} \frac{g_n(\omega )}{\sqrt{1+|n|^2}}e^{in\cdot x}, \end{aligned}$$

where $\{g_n\}_{n \in \mathbb {Z}^2}$ is a sequence of independent standard^5^ complex-valued Gaussian random variables on a probability space $(\Omega , \mathcal {F}, P)$. Namely, functions under $\mu$ are represented by the random Fourier series given in (1.10). Note that the random function *u* in (1.10) is in $H^{s}(\mathbb {T}^2)\setminus L^2(\mathbb {T}^2)$ for any $s < 0$, almost surely. Thus, $\mu$ is a Gaussian probability measure on $H^s(\mathbb {T}^2)$ for any $s< 0$. Moreover, it is easy to see that (1.9) with $\mathcal {H}= H^s(\mathbb {T}^2)$ $Q_s = (\text {Id}-\Delta )^{-1+s}$, $s< 0$, follows from (1.10). Indeed, we have

1.11 $$\begin{aligned} \int _{H^s} \langle f, u \rangle _{H^s} \overline{\langle h, u \rangle }_{H^s} d\mu (u)&= \mathbb {E}\bigg [ \sum _{n \in \mathbb {Z}^2} \frac{\widehat{f}(n) \overline{g_n(\omega )}}{\langle n \rangle ^{1-2s}} \sum _{m \in \mathbb {Z}^2} \frac{\overline{\widehat{h}(m)} g_m(\omega )}{\langle m \rangle ^{1-2s}} \bigg ]\nonumber \\&= \sum _{n \in \mathbb {Z}^2} \frac{\widehat{f}(n)\overline{\widehat{h}(n)}}{\langle n \rangle ^{2-4s}} = \langle Q_s f, h \rangle _{H^s}. \end{aligned}$$

Here, $\langle \,\cdot \, \rangle = (1 + |\cdot |)^\frac{1}{2}$. Note that the second equality in (1.11) holds even for $s\ge 0$. For $s\ge 0$, however, $\mu$ is not a probability measure on $H^s(\mathbb {T}^2)$. Indeed, we have $\mu (L^2(\mathbb {T}^2)) = 0$.

The next step is to make sense of the Gibbs measure $P^{(2m)}_2$ in (1.7). First, let us briefly go over the situation when $d = 1$. In this case, $\mu$ defined by (1.8) is a probability measure on $H^s(\mathbb {T})$, $s < \frac{1}{2}$. Then, it follows from Sobolev’s inequality that $\int _\mathbb {T}|u(x; \omega )|^k dx$ is finite almost surely. Hence, for any $k > 2$, the Gibbs measure:

1.12 $$\begin{aligned} d P^{(k)}_1 = Z^{-1} e^{-\frac{1}{k} \int _{\mathbb {T}} |u|^k dx} d\mu \end{aligned}$$

is a probability measure on $$H^s(\mathbb {T})$$, $$s < \frac{1}{2}$$, absolutely continuous with respect to $$\mu $$. Moreover, by constructing global-in-time dynamics in the support of $$ P^{(k)}_1$$, Bourgain [6] proved that the Gibbs measure $$ P^{(k)}_1$$ is invariant under the dynamics of the defocusing NLS for $$k > 2$$. Here, by invariance, we mean that

1.13 $$\begin{aligned} P^{(k)}_1\big (\Phi (-t) A\big ) = P^{(k)}_1(A) \end{aligned}$$

for any measurable set $A \in \mathcal {B}_{H^s(\mathbb {T})}$ and any $t \in \mathbb {R}$, where $\Phi (t): u_0 \in H^s(\mathbb {T}) \mapsto u(t) = \Phi (t) u_0 \in H^s(\mathbb {T})$ is a well-defined solution map, at least almost surely with respect to $P^{(k)}_1$. McKean [26] gave an independent proof of the invariance of the Gibbs measure when $k = 4$, relying on a probabilistic argument. See Remark 1.8 below for the discussion on the focusing case. Over the recent years, there has been a significant progress in the study of invariant Gibbs measures for Hamiltonian PDEs. See, for example, [6–8, 10, 12, 13, 15, 20, 22, 25, 26, 28, 29, 32, 33, 35, 38, 41–45].

The situation for $d = 2$ is entirely different. As discussed above, the random function *u* in (1.10) is not in $L^2(\mathbb {T}^{2})$ almost surely. This in particular implies that

1.14 $$\begin{aligned} \int _{\mathbb {T}^2} |u(x; \omega )|^k dx = \infty \end{aligned}$$

almost surely for any $k \ge 2$. Therefore, we can not construct a probability measure of the form:

1.15 $$\begin{aligned} d P_2^{(k)} = Z^{-1} e^{-\frac{1}{k} \int _{\mathbb {T}^2} |u|^k dx} d\mu . \end{aligned}$$

Thus, we are required to perform a (Wick) renormalization on the nonlinear part $|u|^{k}$ of the Hamiltonian. This is a well studied subject in the Euclidean quantum field theory, at least in the real-valued setting. See Simon [39] and Glimm-Jaffe [24]. Also, see Da Prato-Tubaro [18] for a concise discussion on $\mathbb {T}^2$, where the Gibbs measures naturally appear in the context of the stochastic quantization equation.

### Wick renormalization

There are different ways to introduce the Wick renormalization. One classical way is to use the Fock-space formalism, where the Wick ordering is given as the reordering of the creation and annihilation operators. See [21, 27, 39] for more details. It can be also defined through the multiple Wiener–Ito integrals. In the following, we directly define it as the orthogonal projection onto the Wiener homogeneous chaoses (see the Wiener–Ito decomposition (2.5) below) by using the Hermite polynomials and the (generalized) Laguerre polynomials, since this allows us to introduce only the necessary objects without introducing cumbersome notations and formalism, making our presentation accessible to readers without prior knowledge in the problem.

Before we study the Wick renormalization for NLS, let us briefly discuss the Wick renormalization on $\mathbb {T}^2$ in the real-valued setting. We refer to [18] for more details. We assume that *u* is real-valued. Then, the random function *u* under $\mu$ in (1.8) is represented by the random Fourier series (1.10) conditioned that $g_{-n} = \overline{g_n}$. Given $N \in \mathbb {N}$, let $\mathbf {P}_N$ be the Dirichlet projection onto the frequencies $\{|n|\le N\}$ and set $u_N = \mathbf {P}_N u$, where *u* is as in (1.10). Note that, for each $x \in \mathbb {T}^2$, the random variable $u_N(x)$ is a mean-zero real-valued Gaussian with variance

1.16 $$\begin{aligned} \sigma _N: = \mathbb {E}[u_N^2(x)] = \sum _{|n|\le N}\frac{1}{1+|n|^2} \sim \log N. \end{aligned}$$

Note that $\sigma _N$ is independent of $x \in \mathbb {T}^2$. Fix an even integer $k \ge 4$. We define the Wick ordered monomial $:\! u_N^k \!:$ by

1.17 $$\begin{aligned} :\! u_N^k \!: \, = H_k(u_N; \sigma _N), \end{aligned}$$

where $H_k(x; \sigma )$ is the Hermite polynomial of degree *k* defined in (2.1). Then, one can show that the limit

1.18 $$\begin{aligned} \int _{\mathbb {T}^2} :\! u^k\!: dx = \lim _{N \rightarrow \infty } \int _{\mathbb {T}^2}:\! u_N^k\!: dx \end{aligned}$$

exists in $L^p( \mu )$ for any finite $p \ge 1$. Moreover, one can construct the Gibbs measure:

$$\begin{aligned} d P_2^{(k)} = Z^{-1} e^{-\frac{1}{k} \int _{\mathbb {T}^2} :u^k : \,dx} d\mu \end{aligned}$$

as the limit of

$$\begin{aligned} d P_{2,N}^{(k)} = Z_N^{-1} e^{-\frac{1}{k} \int _{\mathbb {T}^2} :u_N^k : \, dx} d\mu . \end{aligned}$$

The key ingredients of the proof of the above claims are the Wiener–Ito decomposition of $L^2(H^s(\mathbb {T}^2), \mu )$ for $s< 0$, the hypercontractivity of the Ornstein-Uhlenbeck semigroup, and Nelson’s estimate [30, 31].

For our problem on NLS (1.2), we need to work on complex-valued functions. In the real-valued setting, the Wick ordering was defined by the Hermite polynomials. In the complex-valued setting, we also define the Wick ordering by the Hermite polynomials, but through applying the Wick ordering the real and imaginary parts separately.

Let *u* be as in (1.10). Given $N\in \mathbb {N}$, we define $u_N$ by

$$\begin{aligned} u_N = \mathbf {P}_N u= \sum _{|n|\le N }\widehat{u} (n)e^{in\cdot x}, \end{aligned}$$

where $\mathbf {P}_N$ is the Dirichlet projection onto the frequencies $\{|n|\le N\}$ as above. Then, for $m \in \mathbb {N}$, we define the Wick ordered monomial $:\! |u_N|^{2m} \!:$ by

1.19 $$\begin{aligned} :\! |u_N |^{2m} \!: \,&= \, :\! \big (({{\mathrm{Re\,}}}u_N)^2 + ({{\mathrm{Im\,}}}u_N)^2\big )^m\!: \, \nonumber \\&= \sum _{\ell = 0}^m \begin{pmatrix} m\\ \ell \end{pmatrix} \, :\! ({{\mathrm{Re\,}}}u_N)^{2\ell } \!: \, :\! ({{\mathrm{Im\,}}}u_N)^{2(m - \ell )}\!:. \end{aligned}$$

It turns out, however, that it is more convenient to work with the Laguerre polynomials in the current complex-valued setting; see Sect. 2. Recall that the Laguerre polynomials $L_m(x)$ are defined through the following generating function:

1.20 $$\begin{aligned} G(t, x) : = \frac{1}{1-t} e^{- \frac{tx}{1-t}} = \sum _{m = 0}^\infty t^mL_m(x), \end{aligned}$$

for $|t| < 1$ and $x\in \mathbb {R}$. For readers’ convenience, we write out the first few Laguerre polynomials in the following:

1.21 $$\begin{aligned}&L_0(x) = 1, \qquad L_1(x) = -x+1, \qquad L_2(x) = \tfrac{1}{2}(x^2 -4x + 2), \nonumber \\&L_3(x) = \tfrac{1}{3!}(-x^3 {+} 9 x^2 - 18 x {+}6), \qquad L_4(x) = \tfrac{1}{4!}(x^4 - 16x^3 +72x^2 -96x {+}24). \end{aligned}$$

More generally, the $L_{m}$ are given by the formula

1.22 $$\begin{aligned} L_m(x) = \sum _{\ell = 0}^m \begin{pmatrix} m\\ \ell \end{pmatrix} \frac{(-1)^\ell }{\ell !} x^\ell . \end{aligned}$$

Given $\sigma >0$, we set

1.23 $$\begin{aligned} L_m(x; \sigma ) := \sigma ^m L_m\big (\tfrac{x}{\sigma }\big ). \end{aligned}$$

Note that $L_m(x; \sigma )$ is a homogenous polynomial of degree *m* in *x* and $\sigma$. Then, given $N \in \mathbb {N}$, we can rewrite the Wick ordered monomial $:\! |u_N|^{2m} \!:$ defined in (1.19) as

1.24 $$\begin{aligned} \, :\! |u_N|^{2m} \!: \, = (-1)^m m! \cdot L_m(|u_N|^2; \sigma _N), \end{aligned}$$

where $\sigma _N$ is given by

1.25 $$\begin{aligned} \sigma _N = \mathbb {E}\left[ |u_N(x)|^2\right] = \sum _{|n|\le N}\frac{1}{1+|n|^2} \sim \log N, \end{aligned}$$

independently of $x \in \mathbb {T}^2$. See Lemma 2.1 for the equivalence of (1.19) and (1.24).

For $$N \in \mathbb {N}$$, let

1.26 $$\begin{aligned} G_N(u) = \frac{1}{2m} \int _{\mathbb {T}^2}:\! |\mathbf {P}_N u|^{2m}\!: dx. \end{aligned}$$

Then, we have the following proposition.

#### Proposition 1.1

Let $m \ge 2$ be an integer. Then, $\{G_N(u)\}_{N\in \mathbb {N}}$ is a Cauchy sequence in $L^p( \mu )$ for any $p\ge 1$. More precisely, there exists $C_{m} > 0$ such that

$$\begin{aligned} \Vert G_M(u) - G_N(u) \Vert _{L^p(\mu )} \le C_{m} (p-1)^m\frac{1}{N^\frac{1}{2}} \end{aligned}$$

for any $p \ge 1$ and any $M \ge N \ge 1$.

Proposition 1.1 states that we can define the limit *G*(*u*) as

$$\begin{aligned} G(u)=\frac{1}{2m} \int _{\mathbb {T}^2} :\! |u|^{2m}\!: dx = \lim _{N \rightarrow \infty } G_N(u) = \frac{1}{2m} \lim _{N \rightarrow \infty } \int _{\mathbb {T}^2}:\! |\mathbf {P}_N u|^{2m}\!: dx \end{aligned}$$

and that $G(u) \in L^p( \mu )$ for any finite $p \ge 2$. This allows us to define the Wick ordered Hamiltonian:

1.27 $$\begin{aligned} H_\text {Wick}(u) = \frac{1}{2}\int _{\mathbb {T}^2} |\nabla u|^2dx + \frac{1}{2m} \int _{\mathbb {T}^2} :\! |u|^{2m}\!: dx \end{aligned}$$

for an integer $m \ge 2$. In order to discuss the invariance property of the Gibbs measures, we need to overcome the following two problems.

(i) Define the Gibbs measure of the form
  1.28 $$\begin{aligned} \text {``}d P^{(2m)}_2= Z^{-1} e^{-H_\mathrm{{Wick}}(u) - \frac{1}{2} M(u)} du\text {''} , \end{aligned}$$
  corresponding to the Wick ordered Hamiltonian $H_\mathrm{{Wick}}$.
(ii) Make sense of the following defocusing Wick ordered NLS on $\mathbb {T}^2$:
  1.29 $$\begin{aligned} i \partial _tu + \Delta u = \, :\!|u|^{2(m-1)} u\!: \;, \qquad (t,x) \in \mathbb {R}\times \mathbb {T}^2, \end{aligned}$$
  arising as a Hamiltonian PDE: $\displaystyle \partial _tu = -i \partial _{\overline{u}} H_\text {Wick}$. In particular, we need to give a precise meaning to the Wick ordered nonlinearity $:\!|u|^{2(m-1)} u\!:$.

Let us first discuss Part (i). For $N \in \mathbb {N}$, let

$$\begin{aligned} R_N(u) = e^{-G_{N}(u)} = e^{-\frac{1}{2m} \int _{\mathbb {T}^2} :|u_N|^{2m} : \, dx} \end{aligned}$$

and define the truncated Gibbs measure $P^{(2m)}_{2, N}$ by

1.30 $$\begin{aligned} d P^{(2m)}_{2, N}:= Z_N^{-1} R_N(u) d\mu = Z_N^{-1} e^{-\frac{1}{2m} \int _{\mathbb {T}^2} :| u_N|^{2m} : \, dx} d\mu , \end{aligned}$$

corresponding to the truncated Wick ordered Hamiltonian:

1.31 $$\begin{aligned} H^N_\mathrm{{Wick}}(u) = \frac{1}{2}\int _{\mathbb {T}^2} |\nabla u|^2dx + \frac{1}{2m} \int _{\mathbb {T}^2} :\! | u_N|^{2m}\!: dx. \end{aligned}$$

Note that $P^{(2m)}_{2, N}$ is absolutely continuous with respect to the Gaussian free field $\mu$.

We have the following proposition on the construction of the Gibbs measure $P^{(2m)}_2$ as a limit of $P^{(2m)}_{2, N}$.

#### Proposition 1.2

Let $m \ge 2$ be an integer. Then, $R_N(u) \in L^p(\mu )$ for any $p\ge 1$ with a uniform bound in *N*, depending on $p \ge 1$. Moreover, for any finite $p \ge 1$, $R_N(u)$ converges to some *R*(*u*) in $L^p(\mu )$ as $N \rightarrow \infty$.

In particular, by writing the limit $R(u)\in L^p(\mu )$ as

$$\begin{aligned} R(u) = e^{-\frac{1}{2m} \int _{\mathbb {T}^2} :|u|^{2m} : \, dx}, \end{aligned}$$

Proposition 1.2 allows us to define the Gibbs measure $P^{(2m)}_2$ in (1.28) by

1.32 $$\begin{aligned} d P^{(2m)}_2=Z^{-1} R(u) d\mu = Z^{-1} e^{-\frac{1}{2m} \int _{\mathbb {T}^2} :|u|^{2m} : \,dx} d\mu . \end{aligned}$$

Then, $P^{(2m)}_2$ is a probability measure on $H^s(\mathbb {T}^2)$, $s< 0$, absolutely continuous to the Gaussian field $\mu$. Moreover, $P^{(2m)}_{2, N}$ converges weakly to $P^{(2m)}_2$.

### Invariant dynamics for the Wick ordered NLS

In this subsection, we study the dynamical problem (1.29). First, we consider the Hamiltonian PDE corresponding to the truncated Wick ordered Hamiltonian $H^N_\mathrm{{Wick}}$ in (1.31):

1.33 $$\begin{aligned} i \partial _tu^N + \Delta u^N = \mathbf {P}_N\big (\! :\!|\mathbf {P}_Nu^N|^{2(m-1)} \mathbf {P}_Nu^N\!: \!\big ). \end{aligned}$$

The high frequency part $\mathbf {P}_N^\perp u^N$ evolves according to the linear flow, while the low frequency part $\mathbf {P}_N u^N$ evolves according to the finite dimensional system of ODEs viewed on the Fourier side. Here, $\mathbf {P}_N^\perp$ is the Dirichlet projection onto the high frequencies $\{|n|> N\}$.

Let $\mu = \mu _N \otimes \mu _N^\perp$, where $\mu _N$ and $\mu _N^\perp$ are the marginals of $\mu$ on $E_N = \text {span}\{ e^{in\cdot x}\}_{|n|\le N}$ and $E_N^\perp = \text {span}\{ e^{in\cdot x}\}_{|n|> N}$, respectively. Then, we can write $P^{(2m)}_{2, N}$ in (1.30) as

1.34 $$\begin{aligned} P^{(2m)}_{2, N}= \widehat{P}^{(2m)}_{2, N}\otimes \mu ^\perp _N, \end{aligned}$$

where $\widehat{P}^{(2m)}_{2, N}$ is the finite dimensional Gibbs measure defined by

1.35 $$\begin{aligned} d\widehat{P}^{(2m)}_{2, N} = \widehat{Z}_N^{-1} e^{-\frac{1}{2m} \int _{\mathbb {T}^2} \, :|\mathbf {P}_N u^N|^{2m} : \, dx} d\mu _N. \end{aligned}$$

Then, it is easy to see that $P^{(2m)}_{2, N}$ is invariant under the dynamics of (1.33); see Lemma 5.1 below. In particular, the law of $u^N(t)$ is given by $P^{(2m)}_{2, N}$ for any $t \in \mathbb {R}$.

For $N \in \mathbb {N}$, define $F_N (u)$ by

1.36 $$\begin{aligned} F_N(u) = \mathbf {P}_N\big (\! :\!|\mathbf {P}_Nu|^{{2(m-1)}} \mathbf {P}_Nu\!: \!\big ). \end{aligned}$$

Then, assuming that *u* is distributed according to the Gaussian free field $\mu$ in (1.8), the following proposition lets us make sense of the Wick ordered nonlinearity $:\!|u|^{{2(m-1)}} u\!:$ in (1.29) as the limit of $F_N(u)$.

#### Proposition 1.3

Let $m \ge 2$ be an integer and $s< 0$. Then, $\{F_N(u)\}_{N\in \mathbb {N}}$ is a Cauchy sequence in $L^p(\mu ; H^s(\mathbb {T}^2))$ for any $p\ge 1$. More precisely, given $\varepsilon >0$ with $s + \varepsilon < 0$, there exists $C_{m, s, \varepsilon } > 0$ such that

1.37 $$\begin{aligned} \big \Vert \Vert F_M(u) - F_N(u) \Vert _{H^s}\big \Vert _{L^p(\mu )} \le C_{m, s, \varepsilon } (p-1)^{m- \frac{1}{2}}\frac{1}{N^\varepsilon } \end{aligned}$$

for any $p \ge 1$ and any $M \ge N \ge 1$.

In the real-valued setting, the nonlinearity corresponding to the Wick ordered Hamiltonian is again given by a Hermite polynomial. Indeed, from (1.17), we have

$$\begin{aligned} \tfrac{1}{k} \partial _{u_N}\big (:\! u_N^k \!: \big )= \tfrac{1}{k}\partial _{u_N} H_k(u_N; \sigma _N) = H_{k-1}(u_N; \sigma _N), \end{aligned}$$

since $\partial _xH_k(x; \rho ) = k H_{k-1}(x; \rho )$; see (2.3). The situation is slightly different in the complex-valued setting. In the proof of Proposition 1.3, the generalized Laguerre polynomials $L^{(\alpha )}_m (x)$ with $\alpha = 1$ plays an important role. See Sect. 3.

We denote the limit by $F(u) = \,\, :\! \!|u|^{2(m-1)} u\!:$ and consider the Wick ordered NLS (1.29). When $m= 2$, Bourgain [7] constructed almost sure global-in-time strong solutions and proved the invariance of the Gibbs measure $P^{(4)}_2$ for the defocusing cubic Wick ordered NLS. See Remark 1.7 below. The main novelty in [7] was to construct local-in-time dynamics in a probabilistic manner, exploiting the gain of integrability for the random rough linear solution. By a similar approach, Burq–Tzvetkov [14, 15] constructed almost sure global-in-time strong solutions and proved the invariance of the Gibbs measure for the defocusing subquintic nonlinear wave equation (NLW) posed on the three-dimensional ball in the radial setting.

On the one hand, when $m=2$, there is only an $\varepsilon$-gap between the regularity of the support $H^{s}(\mathbb {T}^2)$, $s< 0$, of the Gibbs measure $P^{(4)}_2$ and the scaling criticality $s = 0$ (and the regularity $s>0$ of the known deterministic local well-posedness [5]). On the other hand, when $m \ge 3$, the gap between the regularity of the Gibbs measure $P^{(2m)}_2$ and the scaling criticality is slightly more than $1 - \frac{1}{m-1} \ge \frac{1}{2}$. At present, it seems very difficult to close this gap and to construct strong solutions even in a probabilistic setting.

In the following, we instead follow the approach presented in the work [12] by the second author with Burq and Tzvetkov. This work, in turn, was motivated by the works of Albeverio–Cruzeiro [1] and Da Prato–Debussche [17] in the study of fluids. The main idea is to exploit the invariance of the truncated Gibbs measures $P^{(2m)}_{2, N}$ for (1.33), then to construct global-in-time *weak* solutions for the Wick ordered NLS (1.29), and finally to prove the invariance of the Gibbs measure $P^{(2m)}_2$ in some mild sense.

Now, we are ready to state our main theorem.

#### Theorem 1.4

Let $m \ge 2$ be an integer. Then, there exists a set $\Sigma$ of full measure with respect to $P^{(2m)}_2$ such that for every $\phi \in \Sigma$, the Wick ordered NLS (1.29) with initial condition $u(0) = \phi$ has a global-in-time solution

$$\begin{aligned} u \in C(\mathbb {R}; H^s(\mathbb {T}^2)) \end{aligned}$$

for any $s < 0$. Moreover, for all $t \in \mathbb {R}$, the law of the random function *u*(*t*) is given by $P^{(2m)}_2$.

There are two components in Theorem 1.4: existence of solutions and invariance of $P^{(2m)}_2$. A precursor to the existence part of Theorem 1.4 appears in [11]. In [11], the second author with Burq and Tzvetkov used the energy conservation and a regularization property under randomization to construct global-in-time solutions to the cubic NLW on $\mathbb {T}^d$ for $d \ge 3$. The main ingredient in [11] is the compactness of the solutions to the approximating PDEs. In order to prove Theorem 1.4, we instead follow the argument in [12]. Here, the main ingredient is the tightness (= compactness) of measures on space-time functions, emanating from the truncated Gibbs measure $P^{(2m)}_{2, N}$ and Skorokhod’s theorem (see Lemma 5.7 below). We point out that Theorem 1.4 states only the existence of a global-in-time solution *u* without uniqueness.

Theorem 1.4 only claims that the law $\mathcal {L}(u(t))$ of the $H^s$-valued random variable *u*(*t*) satisfies

$$\begin{aligned} \mathcal {L}(u(t)) = P^{(2m)}_2\end{aligned}$$

for any $t \in \mathbb {R}$. This implies the invariance property of the Gibbs measure $P^{(2m)}_2$ in some mild sense, but it is weaker than the actual invariance in the sense of (1.13).

In fact, the result of Theorem 1.4 remains true in a more general setting. Let $(\mathcal {M},g)$ be a two-dimensional compact Riemannian manifold without boundary or a bounded domain in $\mathbb {R}^2$. We consider the Eq. (1.1) on $\mathcal {M}$ (when $\mathcal {M}$ is a domain in $\mathbb {R}^2$, we impose the Dirichlet or Neumann boundary condition). Assume that $k=2m$ for some integer $m\ge 2$. In Sect. 4, we prove the analogues of Propositions 1.1, 1.2, and 1.3 in this geometric setting, by incorporating the geometric information such as the eigenfunction estimates. In particular, it is worthwhile to note that the variance parameter $\sigma _N$ in (1.25) now depends on $x \in \mathcal {M}$ in this geometric setting and more care is needed. Once we establish the analogues of Propositions 1.1, 1.2, and 1.3, we can proceed as in the flat torus case. Namely, these propositions allow us to define a renormalized Hamiltonian:

$$\begin{aligned} H_\mathrm{{Wick}}(u) = \frac{1}{2}\int _{\mathcal {M}} |\nabla u|^2dx + \frac{1}{2m} \int _{\mathcal {M}} :\! |u|^{2m}\!: dx, \end{aligned}$$

and a Gibbs measure $P^{(2m)}_2$ as in (1.28). Moreover, we are able to give a sense to NLS with a Wick ordered nonlinearity:

1.38 $$\begin{aligned} \left\{ \begin{array}{l} i \partial _tu + \Delta _g u = : |u|^{2(m-1)}u \! : \\ u|_{t = 0} = \phi , \end{array}\right. \qquad (t,x) \in \mathbb {R}\times \mathcal {M}. \end{aligned}$$

In this general setting, we have the following result.

#### Theorem 1.5

Let $m \ge 2$ be an integer. Then, there exists a set $\Sigma$ of full measure with respect to $P^{(2m)}_2$ such that for every $\phi \in \Sigma$, the Wick ordered NLS (1.38) with initial condition $u(0) = \phi$ has a global-in-time solution

$$\begin{aligned} u \in C(\mathbb {R}; H^s(\mathcal {M})) \end{aligned}$$

for any $s < 0$. Moreover, for all $t \in \mathbb {R}$, the law of the random function *u*(*t*) is given by $P^{(2m)}_2$.

Theorems 1.4 and 1.5 extend [12, Theorem 1.11] for the defocusing Wick ordered cubic NLS ($m = 2$) to all defocusing nonlinearities (all $m \ge 2$). While the main structure of the argument follows that in [12], the main source of challenge for our problem is the more and more complicated combinatorics for higher values of *m*. See Appendix A for an example of an concrete combinatorial argument for $m = 3$ in the case $\mathcal {M}=\mathbb {T}^2$, following the methodology in [7, 12]. In order to overcome this combinatorial difficulty, we introduce the *white noise functional* (see Definition 2.2 below) and avoid combinatorial arguments of increasing complexity in *m*, allowing us to prove Propositions 1.1 and 1.3 in a concise manner. In order to present how we overcome the combinatorial complexity in a clear manner, we decided to first discuss the proofs of Propositions 1.1, 1.2, and 1.3 in the case of the flat torus $\mathbb {T}^2$ (Sects. 2, 3). This allows us to isolate the main idea. We then discuss the geometric component and prove the analogues of Propositions 1.1, 1.2, and 1.3 in a general geometric setting (Sect. 4).

#### Remark 1.6

Our notion of solutions constructed in Theorems 1.4 and 1.5 basically corresponds to that of martingale solutions studied in the field of stochastic PDEs. See, for example, [19].

#### Remark 1.7

Let $m = 2$ and $\mathcal {M}= \mathbb {T}^2$. Then, the Wick ordered NLS (1.29) can be formally written as

1.39 $$\begin{aligned} i \partial _tu + \Delta u = (|u|^2 - 2\sigma _\infty ) u, \end{aligned}$$

where $\sigma _\infty$ is the (non-existent) limit of $\sigma _N \sim \log N$ as $N \rightarrow \infty$.

Given *u* as in (1.10), define , where . Then, it is easy to see that the limit $\theta _\infty := \lim _{N \rightarrow \infty } \theta _N$ exists in $L^p(\mu )$ for any $p \ge 1$. Thus, by setting $v (t) = e^{2 i t \theta _\infty }u (t)$, we can rewrite (1.39) as1.40Note that $\Vert v\Vert _{L^2} = \infty$ almost surely. Namely, (1.40) is also a formal expression for the limiting dynamics. In [7], Bourgain studied (1.40) and proved local well-posedness below $L^2(\mathbb {T}^2)$ in a probabilistic setting.

If *v* is a smooth solution to (1.40), then by setting , we see that *w* is a solution to the standard cubic NLS:

1.41 $$\begin{aligned} i \partial _tw + \Delta w = |w|^2 w. \end{aligned}$$

This shows that the Wick ordered NLS (1.39) and (1.40) are “equivalent” to the standard cubic NLS in the smooth setting. Note that this formal reduction relies on the fact that the Wick ordering introduces only a linear term when $m=2$. For $m \ge 3$, the Wick ordering introduces higher order terms and thus there is no formal equivalence between the standard NLS (1.2) and the Wick ordered NLS (1.29).

#### Remark 1.8

So far, we focused on the defocusing NLS. Let us now discuss the situation in the focusing case:

$$\begin{aligned} i \partial _tu + \Delta u = - |u|^{k-2}u \end{aligned}$$

with the Hamiltonian given by

$$\begin{aligned} H(u) = \frac{1}{2}\int _{\mathbb {T}^d} |\nabla u|^2dx - \frac{1}{k} \int _{\mathbb {T}^d} |u|^k dx. \end{aligned}$$

In the focusing case, the Gibbs measure can be formally written as

$$\begin{aligned} d P^{(k)}_d = Z^{-1} e^{-H(u)} du = Z^{-1} e^{\frac{1}{k} \int _{\mathbb {T}^d} |u|^k dx} d\mu . \end{aligned}$$

The main difficulty is that $\int _{\mathbb {T}^d} |u|^k dx$ is unbounded. When $d = 1$, Lebowitz–Rose–Speer [25] constructed the Gibbs measure $P^{(k)}_1$ for $2< k \le 6$, by adding an extra $L^2$-cutoff. Then, Bourgain  [6] constructed global-in-time flow and proved the invariance of the Gibbs measure for $k \le 6.$ See also McKean [26].

When $$ d= 2$$, the situation becomes much worse. Indeed, Brydges–Slade [9] showed that the Gibbs measure $$P^{(4)}_2$$ for the focusing cubic NLS on $$\mathbb {T}^2$$ can not be realized as a probability measure even with the Wick order nonlinearity and/or with a (Wick ordered) $$L^2$$-cutoff. In [8], Bourgain pointed out that an $$\varepsilon $$-smoothing on the nonlinearity makes this problem well-posed and the invariance of the Gibbs measure may be proven even in the focusing case.

#### Remark 1.9

In a recent paper [37], we also studied the defocusing nonlinear wave equations (NLW) in two spatial dimensions (with an even integer $k = 2m \ge 4$ and $\rho \ge 0$):

1.42 $$\begin{aligned} \left\{ \begin{array}{l} \partial _t^2 u - \Delta _g u + \rho u + u^{k-1} = 0 \\ (u, \partial _tu) |_{t = 0} = (\phi _0, \phi _1), \end{array}\right. \qquad (t,x) \in \mathbb {R}\times \mathcal {M} \end{aligned}$$

and its associated Gibbs measure:

1.43 $$\begin{aligned} dP^{(2m)}_2&= Z^{-1} \exp (- H(u,\partial _tu ))du\otimes d(\partial _tu) \nonumber \\&= Z^{-1} e^{-\frac{1}{2m} \int u^{2m} dx} e^{-\frac{1}{2} \int (\rho u^2 + |\nabla u |^2) dx} du\otimes e^{-\frac{1}{2} \int (\partial _tu)^2} d(\partial _tu). \end{aligned}$$

As in the case of NLS, the Gibbs measure in (1.43) is not well defined in the two spatial dimensions. Namely, one needs to consider the Gibbs measure $P^{(2m)}_2$ associated to the Wick ordered Hamiltonian^6^ as in (1.32) and study the associated dynamical problem given by the following defocusing Wick ordered NLW:

1.44 $$\begin{aligned} \partial _t^2 u - \Delta u + \rho u \, + :\! u^{k-1} \!:\, = 0. \end{aligned}$$

In the case of the flat torus $\mathcal {M}= \mathbb {T}^2$ with $\rho > 0$, we showed that the defocusing Wick ordered NLW (1.44) is almost surely globally well-posed with respect to the Gibbs measure $P^{(2m)}_2$ and that the Gibbs measure $P^{(2m)}_2$ is invariant under the dynamics of (1.44). For a general two-dimensional compact Riemannian manifold without boundary or a bounded domain in $\mathbb {R}^2$ (with the Dirichlet or Neumann boundary condition), we showed that an analogue of Theorem 1.5 (i.e. almost sure global existence and invariance of the Gibbs measure $P^{(2m)}_2$ in some mild sense) holds for (1.44) when $\rho > 0$. In the latter case with the Dirichlet boundary condition, we can also take $\rho = 0$.

In particular, our result on $\mathbb {T}^2$ is analogous to that for the defocusing cubic NLS on $\mathbb {T}^2$ [7], where the main difficulty lies in constructing local-in-time unique solutions almost surely with respect to the Gibbs measure. We achieved this goal for any even $k\ge 4$ by exploiting one degree of smoothing in the Duhamel formulation of the Wick ordered NLW (1.44). As for the Wick ordered NLS (1.29) on $\mathbb {T}^2$, such smoothing is not available and the construction of unique solutions with the Gibbs measure as initial data remains open for the (super)quintic case.

#### Remark 1.10

In [6, 38], Bourgain ($k = 2, 3$) and Richards ($k = 4$) proved invariance of the Gibbs measures for the generalized KdV equation (gKdV) on the circle:

1.45 $$\begin{aligned} \partial _tu + \partial _x^3 u = \pm \partial _x(u^k), \qquad ( t, x) \in \mathbb {R}\times \mathbb {T}. \end{aligned}$$

In [36], the authors and Richards studied the problem for $k \ge 5$. In particular, by following the approach in [12] and this paper, we proved almost sure global existence and invariance of the Gibbs measures in some mild sense analogous to Theorem 1.4 for (i) all $k \ge 5$ in the defocusing case and (ii) $k = 5$ in the focusing case. Note that there is no need to apply a renormalization for constructing the Gibbs measures for this problem since the equation is posed on $\mathbb {T}$. See [6, 25].

This paper is organized as follows. In Sects. 2 and 3, we present the details of the proofs of Propositions 1.1, 1.2, and 1.3 in the particular case when $\mathcal {M}=\mathbb {T}^2$. We then indicate the changes required to treat the general case in Sect. 4. In Sect. 5, we prove Theorems 1.4 and 1.5. In Appendix A, we present an alternative proof of Proposition 1.1 when $m = 3$ in the case $\mathcal {M}= \mathbb {T}^2$, performing concrete combinatorial computations.

## Construction of the Gibbs measures

In this section, we present the proofs of Propositions 1.1 and 1.2 and construct the Gibbs measure $P^{(2m)}_2$ in (1.32). One possible approach is to use the Fock-space formalism in quantum field theory [21, 24, 27, 39]. As mentioned above, however, we present a pedestrian Fourier analytic approach to the problem since we believe that it is more accessible to a wide range of readers. The argument presented in this section and the next section (on Proposition 1.3) follows the presentation in [18] with one important difference; we work in the complex-valued setting and hence we will make use of the (generalized) Laguerre polynomials instead of the Hermite polynomials. Their orthogonal properties play an essential role. See Lemmas 2.4 and 3.2.

### Hermite polynomials, Laguerre polynomials, and Wick ordering

First, recall the Hermite polynomials $H_n(x; \sigma )$ defined through the generating function:

2.1 $$\begin{aligned} F(t, x; \sigma ) : = e^{tx - \frac{1}{2}\sigma t^2} = \sum _{k = 0}^\infty \frac{t^k}{k!} H_k(x;\sigma ) \end{aligned}$$

for $t, x \in \mathbb {R}$ and $\sigma > 0$. For simplicity, we set $F(t, x) : = F(t, x; 1)$ and $H_k(x) : = H_k(x; 1)$ in the following. Note that we have

2.2 $$\begin{aligned} H_k(x, \sigma ) = \sigma ^{\frac{k}{2}}H_k\big (\sigma ^{-\frac{1}{2}}x \big ). \end{aligned}$$

From (2.1), we directly deduce the following recursion relation

2.3 $$\begin{aligned} \partial _{x}H_{k}(x;\sigma )=kH_{k-1}(x;\sigma ), \end{aligned}$$

for all $k\ge 0$. This allows us to compute the $H_{k}$, up to the constant term. The constant term is given by

$$\begin{aligned} H_{2k}(0,\sigma )=(-1)^k (2k-1)!! \, \sigma ^k \qquad \text {and} \qquad H_{2k+1}(0,\sigma )=0, \end{aligned}$$

for all $k\ge 0$, where $(2k-1)!! = (2k-1)(2k-3)\ldots 3\cdot 1 = \frac{(2k)!}{2^k k!}$ and $(-1)!! = 1$ by convention. This can be easily deduced from (2.1) by taking $x=0$. For readers’ convenience, we write out the first few Hermite polynomials in the following:

$$\begin{aligned}&H_0(x; \sigma ) = 1, \qquad H_1(x; \sigma ) = x, \qquad H_2(x; \sigma ) = x^2 - \sigma , \\&H_3(x; \sigma ) = x^3 - 3\sigma x, \qquad H_4(x; \sigma ) = x^4 - 6\sigma x^2 +3\sigma ^2. \end{aligned}$$

The monomial $x^k$ can be expressed in term of the Hermite polynomials:

2.4 $$\begin{aligned} x^k = \sum _{m = 0}^{[\frac{k}{2}]} \begin{pmatrix} k\\ 2m \end{pmatrix} (2m-1)!! \, \sigma ^m H_{k-2m}(x; \sigma ). \end{aligned}$$

Fix $d \in \mathbb {N}$,^7^ let $\mathcal {H}= \mathbb {R}^d$. Then, consider the Hilbert space^8^ $\Gamma _\mathcal {H}= L^2(Q_\mathcal {H}, \mu _d; \mathbb {C})$ endowed with the Gaussian measure $d\mu _d = (2\pi )^{-\frac{d}{2}} \exp (-{|x|^2}/{2})dx$, $x = (x_1, \dots , x_d)\in \mathbb {R}^d$. We define a *homogeneous Wiener chaos of order k* to be an element of the form

$$\begin{aligned} \mathbf{H}_{k}(x) = \prod _{j = 1}^d H_{k_j}(x_j), \end{aligned}$$

where $k= k_1 + \cdots + k_d$ and $H_{k_j}$ is the Hermite polynomial of degree $k_j$ defined in (2.1). Denote by $\Gamma _k(\mathcal {H})$ the closure of homogeneous Wiener chaoses of order *k* under $L^2(\mathbb {R}^d, \mu _d)$. Then, we have the following Wiener–Ito decomposition^9^:

2.5 $$\begin{aligned} L^2(Q_\mathcal {H}, \mu _d; \mathbb {C}) = \bigoplus _{k = 0}^\infty \Gamma _k(\mathcal {H}). \end{aligned}$$

Given a homogeneous polynomial $P_k(x) =P_k(x_1, \dots , x_d)$ of degree *k*, we define *the Wick ordered polynomial* $\, :\!P_k(x)\!\!: \,$ to be its projection onto $\Gamma _k(\mathcal {H})$. In particular, we have $:x_j^k\!: \, = H_{k}(x_j)$ and $:\prod _{j = 1}^d x_j^{k_j}\!:\, = \prod _{j = 1}^d H_{k_j}(x_j)$ with $k= k_1 + \cdots + k_d$.

Now, let *g* be a standard complex-valued Gaussian random variable. Then, *g* can be written as $g = \frac{h_1}{\sqrt{2}}+ i\frac{h_2}{\sqrt{2}}$, where $h_1$ and $h_2$ are independent standard real-valued Gaussian random variables. We investigate the Wick ordering on $|g|^{2m}$ for $m\in \mathbb {N}$, that is, the projection of $|g|^{2m}$ onto $\Gamma _{2m}(\mathcal {H})$. When $m = 1$, $|g|^2 = \frac{1}{2} (h_1^2 + h_2^2)$ is Wick-ordered into

2.6 $$\begin{aligned} :\!|g|^2\!: \, = \tfrac{1}{2} \left( h_1^2 - 1\right) + \tfrac{1}{2} \left( h_2^2-1\right) = |g|^2 - 1. \end{aligned}$$

When $m = 2$, $|g|^4 = \frac{1}{4} (h_1^2+ h_2^2)^2 = \frac{1}{4} (h_1^4 + 2 h_1^2 h_2^2 + h_2^4)$ is Wick-ordered into

$$\begin{aligned} :\!|g|^4\!:\,&= \tfrac{1}{4} \left( h_1^4 -6 h_1^2 + 3\right) + \tfrac{1}{2} \left( h_1^2 - 1\right) \left( h_2^2 - 1\right) + \tfrac{1}{4}\left( h_2^4 -6 h_2^2 + 3\right) \\&= \tfrac{1}{4}\left( h_1^4 + 2 h_1^2 h_2^2 + h_2^4\right) - 2 \left( h_1^2 + h_2^2\right) + 2 \\&= |g|^4 - 4 |g|^2 + 2. \end{aligned}$$

When $m = 3$, a direct computation shows that

$$\begin{aligned} |g|^6 = \frac{1}{8} \left( h_1^2+ h_2^2\right) ^3 = \frac{1}{8}\left( h_1^6 + 3 h_1^4 h_2^2 +3h_1^2 h_2^4 +h_2^6\right) \end{aligned}$$

is Wick-ordered into

$$\begin{aligned} :\!|g|^6\!: \,&= \tfrac{1}{8} H_6(h_1) + \tfrac{3}{8} H_4(h_1) H_2(h_2) + \tfrac{3}{8} H_2(h_1) H_4(h_2) + \tfrac{1}{8} H_6(h_2) \\&= |g|^6 - 9|g|^4 + 18 |g|^2 - 6. \end{aligned}$$

In general, we have

2.7 $$\begin{aligned} :\! |g |^{2m} \!: \,&= \frac{1}{2^m}\sum _{\ell = 0}^m \begin{pmatrix} m\\ \ell \end{pmatrix} H_{2\ell }(h_1 ) H_{2m - 2\ell }(h_2)\nonumber \\&= \sum _{\ell = 0}^m \begin{pmatrix} m\\ \ell \end{pmatrix} H_{2\ell }\left( {{\mathrm{Re\,}}}g; \tfrac{1}{2}\right) H_{2m - 2\ell }\left( {{\mathrm{Im\,}}}g; \tfrac{1}{2}\right) , \end{aligned}$$

where we used (2.2) in the second equality. It follows from the rotational invariance of the complex-valued Gaussian random variable that $:\! |g |^{2m} \!: \, = P_m(|g|^2)$ for some polynomial $P_m$ of degree *m* with the leading coefficient 1. This fact is, however, not obvious from (2.7).

The following lemma shows that the Wick ordered monomials $:\! |g |^{2m} \!:$ can be expressed in terms of the Laguerre polynomials [recall the definition (1.20)].

#### Lemma 2.1

Let $m \in \mathbb {N}$. For a complex valued mean-zero Gaussian random variable *g* with $Var (g) = \sigma >0$, we have

2.8 $$\begin{aligned} :\! |g |^{2m} \!: \,= & {} \sum _{\ell = 0}^m \begin{pmatrix} m\\ \ell \end{pmatrix} H_{2\ell }\left( {{\mathrm{Re\,}}}g; \tfrac{\sigma }{2}\right) H_{2m - 2\ell }\left( {{\mathrm{Im\,}}}g; \tfrac{\sigma }{2}\right) \nonumber \\= & {} (-1)^m m!\cdot L_m(|g|^2; \sigma ). \end{aligned}$$

As a consequence, the Wick ordered monomial $:\! |u_N|^{2m} \!:$ defined in (1.19) satisfies (1.24) for any $N \in \mathbb {N}$.

#### Proof

The first equality follows from (2.7) and scaling with (2.2). Moreover, by scaling with (1.23) and (2.2), we can assume that *g* is a standard complex-valued Gaussian random variable with $g_1 = {{\mathrm{Re\,}}}g$ and $g_2 = {{\mathrm{Im\,}}}g$. Define $\mathfrak {H}_m(|g|^2)$ and $\mathfrak {L}_m(|g|^2)$ by

2.9 $$\begin{aligned} \mathfrak {H}_m(|g|^2)&= \sum _{\ell = 0}^m \begin{pmatrix} m\\ \ell \end{pmatrix} H_{2\ell }\left( g_1; \tfrac{1}{2}\right) H_{2m - 2\ell }\left( g_2 ; \tfrac{1}{2}\right) , \nonumber \\ \mathfrak {L}_m(|g|^2)&= (-1)^m m!\cdot L_m(|g|^2). \end{aligned}$$

Then, (2.8) follows once we prove the following three properties:

2.10 $$\begin{aligned}&\mathfrak {H}_1(|g|^2) = \mathfrak {L}_1(|g|^2) = |g|^2 - 1, \end{aligned}$$

2.11 $$\begin{aligned}&{\left\{ \begin{array}{ll} \phantom {\Big |} \frac{\partial ^2}{\partial g \partial \overline{g}} \mathfrak {H}_m(|g|^2) = m^2 \mathfrak {H}_{m-1} (|g|^2),\\ \phantom {\Big |} \frac{\partial ^2}{\partial g \partial \overline{g}} \mathfrak {L}_m(|g|^2) = m^2 \mathfrak {L}_{m-1}(|g|^2), \end{array}\right. } \end{aligned}$$

2.12 $$\begin{aligned}&\mathbb {E}[\mathfrak {H}_m(|g|^2)] = \mathbb {E}[\mathfrak {L}_m(|g|^2)] = 0, \end{aligned}$$

for all $m \ge 2$. Noting that both $\mathfrak {H}_m(|g|^2)$ and $\mathfrak {L}_m(|g|^2)$ are polynomials in $|g|^2$, the three properties (2.10), (2.11), and (2.12) imply that $\mathfrak {H}_m(|g|^2) = \mathfrak {L}_m(|g|^2)$ for all $m \in \mathbb {N}$.

The first property (2.10) follows from (2.6) and (1.21). Next, we prove (2.11) for $\mathfrak {H}_m(|g|^2)$. From $\partial _{g} = \frac{1}{2}( \partial _{g_1} - i \partial _{g_2})$ and $\partial _{\overline{g}} = \frac{1}{2}( \partial _{g_1} + i \partial _{g_2})$, we have

$$\begin{aligned} \frac{\partial ^2}{\partial g \partial \overline{g}} = \frac{1}{4} \Delta _{g_1, g_2}, \end{aligned}$$

where $\Delta _{g_1, g_2}$ denotes the usual Laplacian on $\mathbb {R}^2$ in the variables $(g_1, g_2)$. Then, recalling that $\partial _xH_k(x; \sigma ) = k H_{k-1}(x; \sigma )$, we have

$$\begin{aligned} \frac{\partial ^2}{\partial g \partial \overline{g}}&\mathfrak {H}_m(|g|^2) = \frac{1}{4} \Delta _{g_1, g_2} \mathfrak {H}_m(|g|^2)\nonumber \\&= \frac{1}{4} \sum _{\ell = 1}^m \begin{pmatrix} m\\ \ell \end{pmatrix} 2\ell (2\ell -1) H_{2\ell -2}\left( g_1; \tfrac{1}{2}\right) H_{2m - 2\ell }\left( g_2 ; \tfrac{1}{2}\right) \nonumber \\&\qquad + \frac{1}{4} \sum _{\ell = 0}^{m-1} \begin{pmatrix} m\\ \ell \end{pmatrix} (2m -2\ell ) (2m - 2\ell - 1) H_{2\ell }\left( g_1; \tfrac{1}{2}\right) H_{2m - 2\ell - 2}\left( g_2 ; \tfrac{1}{2}\right) \nonumber \\&= m^2 \sum _{\ell = 0}^{m-1} \begin{pmatrix} m - 1\\ \ell \end{pmatrix} H_{2\ell }\left( g_1; \tfrac{1}{2}\right) H_{2(m-1) - 2\ell }\left( g_2 ; \tfrac{1}{2}\right) . \nonumber \end{aligned}$$

As for the second identity in (2.11), thanks to the formula (1.22), we get

$$\begin{aligned} \frac{\partial ^2}{\partial g \partial \overline{g}} \mathfrak {L}_m(|g|^2)&= \frac{(-1)^m m!}{4} \sum _{\ell = 0}^m \begin{pmatrix} m\\ \ell \end{pmatrix} \frac{(-1)^\ell }{\ell !} \Delta _{g_1, g_2} \left( g_1^2 + g_2^2\right) ^\ell \\&= (-1)^{m-1} m! \sum _{\ell = 1}^m \begin{pmatrix} m\\ \ell \end{pmatrix} \frac{(-1)^{\ell -1}}{\ell !} \ell ^2 |g|^{2(\ell -1)} = m^2 \mathfrak {L}_{m-1}(|g|^2). \end{aligned}$$

This proves (2.11). The property (2.12) follows from (i) independence of $g_1$ and $g_2$ together with the orthogonality of $H_k(x)$ and the constant function 1 under $e^{-x^2} dx$ and (ii) the orthogonality of $L_m(x)$ and the constant function 1 under $\mathbf 1_{\mathbb {R}_+}e^{-x} dx$

Let *u* be as in (1.10). Fix $x \in \mathbb {T}^2$. Letting $\widetilde{g}_n = g_n e^{in\cdot x}$, we see that $\{\widetilde{g}_n\}_{n \in \mathbb {N}}$ is a sequence of independent standard complex-valued Gaussian random variables. Then, given $N \in \mathbb {N}$, ${{\mathrm{Re\,}}}u_N(x)$ and ${{\mathrm{Im\,}}}u_N(x)$ are mean-zero real-valued Gaussian random variables with variance $\frac{\sigma _N}{2}$, while $u_N(x)$ is a mean-zero complex-valued Gaussian random variable with variance $\sigma _N$, Then, it follows from (1.19) with (1.17) and (2.8) that

$$\begin{aligned} :\! |u_N(x) |^{2m} \!: \,&= \sum _{\ell = 0}^m \begin{pmatrix} m\\ \ell \end{pmatrix} H_{2\ell }\left( {{\mathrm{Re\,}}}u(x); \tfrac{\sigma _N}{2}\right) H_{2m - 2\ell }\left( {{\mathrm{Im\,}}}u(x) ; \tfrac{\sigma _N}{2}\right) \nonumber \\&= (-1)^m m!\cdot L_m\left( |u_N(x)|^2; \sigma _N\right) , \end{aligned}$$

verifying (1.24). This proves the second claim in Lemma 2.1. $\square$

### White noise functional

Next, we define the white noise functional. Let $w(x;\omega )$ be the mean-zero complex-valued Gaussian white noise on $\mathbb {T}^2$ defined by

$$\begin{aligned} w(x;\omega ) = \sum _{n\in \mathbb {Z}^2} g_n(\omega ) e^{in\cdot x}. \end{aligned}$$

#### Definition 2.2

The white noise functional $W_{(\cdot )}: L^2(\mathbb {T}^2) \rightarrow L^2(\Omega )$ is defined by

2.13 $$\begin{aligned} W_f (\omega ) = \langle f, w(\omega ) \rangle _{L^2_x} = \sum _{n \in \mathbb {Z}^2} \widehat{f}(n) \overline{g_n}(\omega ) \end{aligned}$$

for a function $$f \in L^2(\mathbb {T}^2)$$.

Note that this is basically the periodic and higher dimensional version of the classical Wiener integral $\int _a^b f dB$. It can also be viewed as the Gaussian process indexed by $f \in L^2(\mathbb {T}^2)$. See [39, Model 1 on p. 19 and Model 3 on p. 21]. For each $f \in L^2(\mathbb {T}^2)$, $W_f$ is a complex-valued Gaussian random variable with mean 0 and variance $\Vert f\Vert _{L^2}^2$. Moreover, we have

$$\begin{aligned} E\big [ W_f \overline{W_h} ] = \langle f, h \rangle _{L^2_x}\end{aligned}$$

for $f, h \in L^2(\mathbb {T}^2)$. In particular, the white noise functional $W_{(\cdot )}: L^2(\mathbb {T}^2) \rightarrow L^2(\Omega )$ is an isometry.

#### Lemma 2.3

Given $f \in L^2(\mathbb {T}^2)$, we have

2.14 $$\begin{aligned} \int _{\Omega } e^{{{\mathrm{Re\,}}}W_f(\omega )} dP(\omega ) = e^{\frac{1}{4}\Vert f\Vert _{L^2}^2}. \end{aligned}$$

#### Proof

Noting that ${{\mathrm{Re\,}}}g_n$ and ${{\mathrm{Im\,}}}g_n$ are mean-zero real-valued Gaussian random variables with variance $\frac{1}{2}$, it follows from (2.13) that

$$\begin{aligned} \int _{\Omega } e^{{{\mathrm{Re\,}}}W_f(\omega )} dP(\omega ) =&\prod _{n \in \mathbb {Z}} \frac{1}{\pi } \int _\mathbb {R}e^{{{\mathrm{Re\,}}}\widehat{f}(n) {{\mathrm{Re\,}}}g_n - ({{\mathrm{Re\,}}}g_n)^2} d {{\mathrm{Re\,}}}g_n\\&\qquad \times \int _\mathbb {R}e^{ {{\mathrm{Im\,}}}\widehat{f}(n) {{\mathrm{Im\,}}}g_n - ({{\mathrm{Im\,}}}g_n)^2} d {{\mathrm{Im\,}}}g_n \\ =&e^{\frac{1}{4}\Vert f\Vert _{L^2}^2}. \end{aligned}$$

$$\square $$

The following lemma on the white noise functional and the Laguerre polynomials plays an important role in our analysis. In the following, we present an elementary proof, using the generating function *G* in (1.20). See also Folland [23].

#### Lemma 2.4

Let $f, h \in L^2(\mathbb {T}^{2})$ such that $\Vert f\Vert _{L^2} = \Vert h\Vert _{L^2} = 1$. Then, for $k, m \in \mathbb {Z}_{\ge 0}$, we have

2.15 $$\begin{aligned} \mathbb {E}\big [ L_k(|W_f|^2)L_m(|W_h|^2)\big ] = \delta _{km} |\langle f, h \rangle |^{2k}. \end{aligned}$$

Here, $\delta _{km}$ denotes the Kronecker delta function.

First, recall the following identity:

2.16 $$\begin{aligned} e^\frac{u^2}{2} = \frac{1}{\sqrt{2\pi }} \int _\mathbb {R}e^{xu - \frac{x^2}{2}} dx. \end{aligned}$$

Indeed, we used a rescaled version of (2.16) in the proof of Lemma 2.3.

#### Proof of Lemma 2.4

Let *G* be as in (1.20). Then, for any $$ -1< t, s <0$$, from (2.16) and Lemma 2.3, we have

$$\begin{aligned}&\int _{\Omega } G\left( t, |W_f(\omega )|^2\right) G\left( s, |W_h(\omega )|^2\right) dP(\omega )\\&\quad =\frac{1}{1-t}\frac{1}{1-s} \int _\Omega e^{-\frac{t}{1-t}|W_f|^2 - \frac{s}{1-s}|W_h|^2} dP(\omega )\nonumber \\&\quad = \frac{1}{1-t}\frac{1}{1-s} \frac{1}{4\pi ^2} \int _{\mathbb {R}^4} e^{-\frac{x_1^2 + x_2^2 + y_1^2 + y_2^2}{2}}\nonumber \\&\qquad \times \int _\Omega \exp \Big ( {{\mathrm{Re\,}}}W_{ \sqrt{\frac{-2t}{1-t}}(x_1 -i x_2 ) f + \sqrt{\frac{-2s}{1-s}}(y_1 -i y_2) h}\Big ) dP dx_1dx_2dy_1dy_2 \nonumber \\&\quad = \frac{1}{1-t}\frac{1}{1-s} \frac{1}{4\pi ^2} \int _{\mathbb {R}^4} e^{-\frac{x_1^2 + x_2^2 }{2(1-t)}-\frac{y_1^2 + y_2^2 }{2(1-s)}} \nonumber \\&\qquad \times e^{\frac{1}{2} {{\mathrm{Re\,}}}\big ( \sqrt{\frac{-2t}{1-t}} \sqrt{\frac{-2s}{1-s}}(x_1 -i x_2 ) (y_1 + i y_2)\langle f, h \rangle \big )} dx_1dx_2dy_1dy_2 \end{aligned}$$

By a change of variables and applying (2.16), we have

2.17 $$\begin{aligned}&= \frac{1}{4\pi ^2} \int _{\mathbb {R}^2} e^{-\frac{ y_1^2 + y_2^2}{2}} \int _\mathbb {R}e^{ \sqrt{ts} (y_1 {{\mathrm{Re\,}}}\langle f, h \rangle - y_2 {{\mathrm{Im\,}}}\langle f, h \rangle )x_1 - \frac{x_1^2}{2}} dx_1 \nonumber \\&\quad \times \int _\mathbb {R}e^{ \sqrt{ts} (y_2 {{\mathrm{Re\,}}}\langle f, h \rangle + y_1 {{\mathrm{Im\,}}}\langle f, h \rangle )x_2- \frac{x_2^2}{2}} dx_2 dy_1dy_2 \nonumber \\&= \frac{1}{2\pi } \int _{\mathbb {R}^2} e^{-\frac{ y_1^2 + y_2^2}{2}} e^{ \frac{1}{2} ts |\langle f, h \rangle |^2 (y_1^2 + y_2^2) }dy_1dy_2 \nonumber \\&= \frac{1}{1-ts |\langle f, h \rangle |^2} = \sum _{k = 0}^\infty t^ks^k |\langle f, h \rangle |^{2k}. \end{aligned}$$

In the second to the last equality, we used the fact that $\frac{1}{2} ts |\langle f, h \rangle |^2 < \frac{1}{2}$. Hence, it follows from (1.20) and (2.17) that

$$\begin{aligned} \sum _{k = 0}^\infty t^ks^k |\langle f, h \rangle |^{2k} = \sum _{k, m = 0}^\infty t^ks^m \int _\Omega L_k\left( |W_f(\omega )|^2\right) L_m\left( |W_h(\omega )|^2\right) dP(\omega ). \end{aligned}$$

By comparing the coefficients of $t^ks^m$, we obtain (2.15). $\square$

Now, we are ready to make sense of the nonlinear part of the Wick ordered Hamiltonian $H_\mathrm{{Wick}}$ in (1.27). We first present the proof of Proposition 1.1 for $p = 2$. Recall that

$$\begin{aligned} G_N(u) = \frac{1}{2m} \int _{\mathbb {T}^2}:\! |\mathbf {P}_N u|^{2m}\!: dx. \end{aligned}$$

Then, we have the following convergence property of $G_N(u)$ in $L^2(\mu )$.

#### Lemma 2.5

Let $m \ge 2$ be an integer. Then, $\{G_N(u)\}_{N\in \mathbb {N}}$ is a Cauchy sequence in $L^2(H^s(\mathbb {T}^2), \mu )$. More precisely, there exists $C_{m} > 0$ such that

2.18 $$\begin{aligned} \Vert G_M(u) - G_N(u) \Vert _{L^2(\mu )} \le \frac{C_m}{N^\frac{1}{2}} \end{aligned}$$

for any $$ M \ge N \ge 1$$.

Given $N \in \mathbb {N}$, let $\sigma _N$ be as in (1.25). For *fixed* $x \in \mathbb {T}^{2}$ and $N \in \mathbb {N}$, we define

2.19 $$\begin{aligned} \eta _N(x) (\cdot )&:= \frac{1}{\sigma _N^\frac{1}{2}} \sum _{ |n| \le N} \frac{\overline{e_n(x)}}{\sqrt{1+ |n|^2}}e_n(\cdot ), \end{aligned}$$

2.20 $$\begin{aligned} \gamma _N (\cdot )&:= \sum _{ |n| \le N} \frac{1}{1+ |n|^2}e_n(\cdot ), \end{aligned}$$

where $e_n(y) = e^{in\cdot y}$. Note that

2.21 $$\begin{aligned} \Vert \eta _N(x)\Vert _{L^2(\mathbb {T}^{2})} = 1 \end{aligned}$$

for all (fixed) $x \in \mathbb {T}^{2}$ and all $N \in \mathbb {N}$. Moreover, we have

2.22 $$\begin{aligned} \langle \eta _M(x), \eta _N(y) \rangle _{L^2(\mathbb {T}^{2})} = \frac{1}{\sigma _M^\frac{1}{2}\sigma _N^\frac{1}{2}} \gamma _N(y-x) = \frac{1}{\sigma _M^\frac{1}{2}\sigma _N^\frac{1}{2}} \gamma _N(x-y), \end{aligned}$$

for fixed $x, y\in \mathbb {T}^{2}$ and $N, M \in \mathbb {N}$ with $M\ge N$.

#### Proof of Lemma 2.5

Let $m \ge 2$ be an integer. Given $N \in \mathbb {N}$ and $x \in \mathbb {T}^{2}$, it follows from (1.10), (2.13), and (2.19) that

2.23 $$\begin{aligned} u_N (x) = \sigma _N^\frac{1}{2}\frac{u_N(x)}{\sigma _N^\frac{1}{2}} = \sigma _N^{\frac{1}{2}} \overline{W_{ {\eta _N(x)}}}. \end{aligned}$$

Then, from (1.24) and (2.23), we have

2.24 $$\begin{aligned} :\! |u_N|^{2m} \!: \, = (-1)^m m! \sigma ^m_NL_m\bigg (\frac{| u_N|^2}{\sigma _N}\bigg ) \, = (-1)^m m! \sigma _N^m L_m\big (\big |W_{ {\eta _N(x)}}\big |^2\big ). \end{aligned}$$

From  (2.24), Lemma 2.4, and (2.22), we have

2.25 $$\begin{aligned} (2m)^{2} \Vert G_M(u) - G_N(u) \Vert _{L^2(\mu )}^2&= (m!)^2 \int _{\mathbb {T}^2_x\times \mathbb {T}^2_y} \int _{\Omega } \Big [ \sigma _M^{2m} L_m\left( \big |W_{ {\eta _M(x)}}\big |^2\right) L_m\big (\big |W_{ {\eta _M(y)}}\big |^2\big ) \nonumber \\&\qquad - \sigma _M^{m}\sigma _N^{m} L_m\left( \big |W_{ {\eta _M(x)}}\big |^2\right) L_m\left( \big |W_{ {\eta _N(y)}}\big |^2\right) \nonumber \\&\qquad - \sigma _M^{m}\sigma _N^{m} L_m\left( \big |W_{ {\eta _N(x)}}\big |^2\right) L_m\big (\big |W_{ {\eta _M(y)}}\big |^2\big ) \nonumber \\&\qquad + \sigma _N^{2m} L_m\left( \big |W_{ {\eta _N(x)}}\big |^2\right) L_m\left( \big |W_{{\eta _N(y)}}\big |^2\right) \Big ] dP dx dy \nonumber \\&= (m!)^2 \int _{\mathbb {T}^2_x\times \mathbb {T}^2_y} \big [ (\gamma _M(x-y))^{2m} - (\gamma _N(x-y))^{2m}\big ] dx dy \nonumber \\&= (m!)^2 \int _{\mathbb {T}^2} \big [ (\gamma _M(x))^{2m} - (\gamma _N(x))^{2m}\big ] dx \nonumber \\&\le C_m \int _{\mathbb {T}^2} \big |\gamma _M(x)-\gamma _N(x)\big | \cdot \big [|\gamma _M(x)|^{2m-1} \nonumber \\&\qquad + |\gamma _N(x)|^{2m-1}\big ] dx . \end{aligned}$$

In the second equality, we used the fact that $\gamma _N$ is a real-valued function.

From (2.20), we have

2.26 $$\begin{aligned} \big \Vert \gamma _M-\gamma _N\big \Vert _{L^2} = \left( \sum _{N < |n|\le M} \frac{1}{(1+|n|^2)^2}\right) ^\frac{1}{2} \lesssim \frac{1}{N}. \end{aligned}$$

By Hausdorff-Young’s inequality, we have

2.27 $$\begin{aligned} \big \Vert |\gamma _N|^{2m-1}\big \Vert _{L^2} = \Vert \gamma _N\Vert _{L^{4m-2}}^{2m-1} \le \left( \sum _{|n|\le N} \frac{1}{(1+|n|^2)^{\frac{4m-2}{4m-3}}}\right) ^{\frac{4m-3}{2}} \le C_m < \infty \end{aligned}$$

uniformly in $N \in \mathbb {N}$. Then, (2.18) follows from (2.25), (2.26), and (2.27). $\square$

### Wiener chaos estimates

In this subsection, we complete the proof of Proposition  1.1. Namely, we upgrade (2.18) in Lemma 2.5 to any finite $p \ge 2$. Our main tool is the following Wiener chaos estimate (see [39, Theorem I.22]).

#### Lemma 2.6

Let $\{ g_n\}_{n \in \mathbb {N}}$ be a sequence of independent standard real-valued Gaussian random variables. Given $k \in \mathbb {N}$, let $\{P_j\}_{j \in \mathbb {N}}$ be a sequence of polynomials in $\bar{g} = \{ g_n\}_{n \in \mathbb {N}}$ of degree at most *k*. Then, for $p \ge 2$, we have

2.28 $$\begin{aligned} \bigg \Vert \sum _{j \in \mathbb {N}} P_j(\bar{g}) \bigg \Vert _{L^p(\Omega )} \le (p-1)^\frac{k}{2} \bigg \Vert \sum _{j \in \mathbb {N}} P_j(\bar{g}) \bigg \Vert _{L^2(\Omega )}. \end{aligned}$$

Observe that the estimate (2.28) is independent of $d \in \mathbb {N}$. By noting that $P_j(\bar{g})\in \bigoplus _{\ell = 0}^k \Gamma _\ell (\mathcal {H})$, this lemma follows as a direct corollary to the hypercontractivity of the Ornstein-Uhlenbeck semigroup due to Nelson [30].

We are now ready to present the proof of Proposition 1.1.

#### Proof of Proposition 1.1

Let $m \ge 2$ be an integer. For $1\le p \le 2$, Proposition 1.1 follows from Lemma 2.5. In the following, we consider the case $p > 2$. From (1.22), (1.24), and (1.26), we have

$$\begin{aligned} G_M(u) - G_N(u) =\frac{ (-1)^m m!}{2m} \sum _{\ell = 1}^m \begin{pmatrix} m\\ \ell \end{pmatrix} \frac{(-1)^\ell }{\ell !} \Sigma _{\ell }. \end{aligned}$$

Here, $\Sigma _\ell$ is given by

$$\begin{aligned} \Sigma _{\ell } = \frac{ \sigma ^{m}_{M}}{\sigma ^{\ell }_{M}}\sum _{\begin{array}{c} \Gamma _{2\ell }(0)\\ |n_j| \le M \end{array}}\prod _{j = 1}^{2\ell } \frac{g^*_{n_j}}{\sqrt{1+|n_j|^2}} - \frac{ \sigma ^{m}_{N}}{\sigma ^{\ell }_{N}}\sum _{\begin{array}{c} \Gamma _{2\ell }(0)\\ |n_j| \le N \end{array}} \prod _{j = 1}^{2\ell } \frac{g^*_{n_j}}{\sqrt{1+|n_j|^2}},\end{aligned}$$

where $\Gamma _k$ and $g^*_{n_j}$ are defined by

2.29 $$\begin{aligned}&\Gamma _k(n) = \{ (n_1, \dots , n_{k}) \in (\mathbb {Z}^2)^{k}: n_1 - n_2 + \cdots +(-1)^k n_{k} = n\}, \end{aligned}$$

2.30 $$\begin{aligned}&g^*_{n_j} = {\left\{ \begin{array}{ll} g_{n_j} &{} \quad \text {if}\, j\, \text {is odd,} \\ \overline{g_{n_j}} &{} \quad \text {if}\, j\, \text {is even.} \end{array}\right. } \end{aligned}$$

Noting that $\Sigma _{\ell }$ is a sum of polynomials of degree $2\ell$ in $\{g_n\}_{n \in \mathbb {Z}^2}$, Proposition 1.1 follows from Lemmas 2.5 and 2.6. $\square$

### Nelson’s estimate

In this subsection, we prove Proposition 1.2. Our main tool is the so-called Nelson’s estimate, i.e. in establishing an tail estimate of size $\lambda >0$, we divide the argument into low and high frequencies, depending on the size of $\lambda$. See (2.32) and (2.34). What plays a crucial role here is the defocusing property of the Hamiltonian and the logarithmic upper bound on $-G_N(u)$, which we discuss below.

For each $m \in \mathbb {N}$, there exists finite $a_m >0$ such that $(-1)^m L_m(x^2) \ge - a_m$ for all $x \in \mathbb {R}$. Then, it follows from  (1.23), (1.24), (1.25), and (1.26) that there exists some finite $b_m > 0$ such that

2.31 $$\begin{aligned} - G_N(u) = - \frac{1}{2m} \int _{\mathbb {T}^2}:\! |\mathbf {P}_N u|^{2m}\!: dx \le b_m (\log N)^{m} \end{aligned}$$

for all $N\ge 1$. Namely, while $G_N(u)$ is not sign definite, $-G_N(u)$ is bounded from above by a power of $\log N$. This is where the defocusing property of the Eq. (1.33) plays an essential role.

#### Proof of Proposition 1.2

Let $m \ge 2$ be an integer. It follows from Proposition 1.1 that the following tail estimate holds: there exist $c_{m,p}, C_m > 0$ such that

2.32 $$\begin{aligned} \mu \big (p | G_M(u) - G_N(u)| > \lambda \big ) \le C_m e^{ - c_{m, p} N^\frac{1}{2m}\lambda ^\frac{1}{m}} \end{aligned}$$

for all $M \ge N \ge 1$, $p \ge 1$, and all $\lambda > 0$. See, for example, [44, Lemma 4.5].

We first show that $R_N(u)=e^{- G_{N}(u)}$ is in $L^p(\mu )$ with a uniform bound in *N*. We have

$$\begin{aligned} \Vert R_{N}(u)\Vert ^{p}_{L^{p}(\mu )}&= \int _{H^{s}} e^{-pG_{N}(u)}d\mu (u)\\&=\int _{0}^{\infty }\mu (e^{-pG_{N}(u)}> \alpha )d \alpha \\&\le 1+\int _{1}^{\infty }\mu (-pG_{N}(u)>\log \alpha )d \alpha . \end{aligned}$$

Hence, it suffices to show that there exist $C,\delta >0$ such that

2.33 $$\begin{aligned} \mu (-pG_{N}(u)>\log \alpha )\le C\alpha ^{-(1+\delta )} \end{aligned}$$

for all $\alpha > 1$ and $N \in \mathbb {N}$. Given $\lambda =\log \alpha > 0$, choose $N_0\in \mathbb {R}$ such that $\lambda = 2 p b_m (\log N_0)^{m}$. Then, it follows from (2.31) that

2.34 $$\begin{aligned} \mu \big ( -p G_N(u) > \lambda \big ) = 0 \end{aligned}$$

for all $$N < N_0$$. For $$N \ge N_0$$, it follows from (2.31) and (2.32) that there exist $$\delta _{m, p}>0$$ and $$ C_{m, p}> 0$$ such that

2.35 $$\begin{aligned} \mu \big ( -p G_N(u)> \lambda \big )&\le \mu \big ( -p G_N(u) + p G_{N_0}(u)> \lambda -p b_m (\log N_0)^{m} \big )\nonumber \\&\le \mu \big ( -p G_N(u) + p G_{N_0}(u) > \tfrac{1}{2}\lambda \big )\nonumber \\&\le C_m e^{ - c'_{m, p} N_0^\frac{1}{2m}\lambda ^\frac{1}{m}} = C_m e^{ - c'_{m, p} \lambda ^\frac{1}{m} e^{\widetilde{c}_m \lambda ^\frac{1}{m}}}\nonumber \\&\ll C_{m, p} e^{-{(1+ \delta _{m, p})\lambda }} \end{aligned}$$

for all $N \ge N_0$. This shows that (2.33) is satisfied in this case as well. Hence, we have $R_N(u) \in L^p(\mu )$ with a uniform bound in *N*, depending on $p \ge 1$.

By (2.32), $G_{N}(u)$ converges to *G*(*u*) in measure with respect to $\mu$. Then, as a composition of $G_N(u)$ with a continuous function, $R_{N}(u)=e^{-G_{N}(u)}$ converges to $R(u):=e^{-G(u)}$ in measure with respect to $\mu$. In other words, given $\varepsilon > 0$, defining $A_{N, \varepsilon }$ by

$$\begin{aligned} A_{N,\varepsilon }=\big \{ \, |R_{N}(u)-R(u)| \le \varepsilon \,\big \}, \end{aligned}$$

we have $\mu ({A^{c}_{N,\varepsilon }})\rightarrow 0$, as $N\rightarrow \infty$. Hence, by Cauchy–Schwarz inequality and the fact that $\Vert R\Vert _{L^{2p}}, \Vert R_{N}\Vert _{L^{2p}}\le C_{p}$ uniformly in $N \in \mathbb {N}$, we obtain

$$\begin{aligned} \Vert R-R_{N}\Vert _{L^{p}(\mu )}&\le \Vert (R-R_{N})\mathbf{1}_{A_{N,\varepsilon }}\Vert _{L^{p}(\mu )}+\Vert (R-R_{N})\mathbf{1}_{{A^{c}_{N,\varepsilon }}}\,\Vert _{L^{p}(\mu )}\\&\le \varepsilon \big (\,\mu (A_{N,\varepsilon }\,)\big )^{\frac{1}{p}}+\Vert R-R_{N} \Vert _{L^{2p}(\mu )} \big (\,\mu ({A^{c}_{N,\varepsilon }})\,\big )^{\frac{1}{2p}}\le C\varepsilon , \end{aligned}$$

for all sufficiently large *N*. This completes the proof of Proposition 1.2. $\square$

## On the Wick ordered nonlinearity

In this section, we present the proof of Proposition 1.3. The main idea is similar to that in Sect. 2 but, this time, we will make use of the generalized Laguerre functions $L_m^{(\alpha )}(x)$. The generalized Laguerre polynomials $L^{(\alpha )}_m(x)$ are defined through the following generating function:

3.1 $$\begin{aligned} G_\alpha (t, x) : = \frac{1}{(1-t)^{\alpha +1}} e^{- \frac{tx}{1-t}} = \sum _{m = 0}^\infty t^mL_m^{(\alpha )}(x), \end{aligned}$$

for $|t| < 1$ and $x\in \mathbb {R}$. From (3.1), we obtain the following differentiation rule; for $\ell \in \mathbb {N}$,

3.2 $$\begin{aligned} \frac{d^\ell }{dx^\ell } L^{(\alpha )}_m(x) = (-1)^\ell L^{(\alpha +\ell )}_{m-\ell }(x). \end{aligned}$$

Given $N \in \mathbb {N}$, let $u_N = \mathbf {P}_N$, where *u* is as in (1.10). Let $m \ge 2$ be an integer. Then, from (1.36), (1.24), (1.23), and (3.2), we have

3.3 $$\begin{aligned} F_N(u)&= \mathbf {P}_N\big (\! :\!|\mathbf {P}_N u|^{2(m-1)} \mathbf {P}_Nu\!: \!\big ) = (-1)^m m! \sigma _N^m \cdot \tfrac{1}{m}\mathbf {P}_N \partial _{\overline{u}_{N}} \Big \{L_m\Big (\tfrac{|u_N|^2}{\sigma _N}\Big )\Big \}\nonumber \\&= (-1)^{m+1} (m-1)! \sigma _N^{m-1} \cdot \mathbf {P}_N \Big \{ L_{m-1}^{(1)}\Big (\tfrac{|u_N|^2}{\sigma _N}\Big ) u_N \Big \}. \end{aligned}$$

### Remark 3.1

Here, $\partial _{\overline{u}}$ denotes the usual differentiation in $\overline{u}$ viewing *u* and $\overline{u}$ as independent variables. This is not to be confused with $\frac{\partial H}{\partial \overline{u}}$ in (1.3). Note that $\frac{\partial H}{\partial \overline{u}}$ in (1.3) comes from the symplectic structure of NLS and the Gâteaux derivative of *H*. More precisely, we can view the dynamics of NLS (1.2) as a Hamiltonian dynamics with the symplectic space $L^2(\mathbb {T}^2)$ and the symplectic form $\displaystyle \omega (f, g) = {{\mathrm{Im\,}}}\int f(x) \overline{g(x)} dx$. Then, we define $\frac{\partial H}{\partial \overline{u}}$ by

$$\begin{aligned} dH|_u(\phi ) = \omega \Big (\phi , -i \tfrac{\partial H}{\partial \overline{u}}\Big ), \end{aligned}$$

where $dH|_u(\phi )$ is the the Gâteaux derivative given by $dH|_u(\phi ) = \frac{d}{d\varepsilon }H(u + \varepsilon \phi )\big |_{\varepsilon = 0}$.

The following lemma is an analogue of Lemma 2.4 for the generalized Laguerre polynomials $L^{(1)}_m(x)$ and plays an important role in the proof of Proposition 1.3.

### Lemma 3.2

Let $f, h \in L^2(\mathbb {T}^{2})$ such that $\Vert f\Vert _{L^2} = \Vert h\Vert _{L^2} = 1$. Then, for $k, m \in \mathbb {Z}_{\ge 0}$, we have

3.4 $$\begin{aligned} \mathbb {E}\Big [ L^{(1)}_k(|W_f|^2)W_f \overline{L^{(1)}_m(|W_h|^2)W_h}\Big ] = \delta _{km}( k+1) |\langle f, h \rangle |^{2k}\langle f, h \rangle . \end{aligned}$$

Here, $\delta _{km}$ denotes the Kronecker delta function.

Besides (2.16), we will use the following identity:

3.5 $$\begin{aligned} u e^\frac{u^2}{2} = \frac{1}{\sqrt{2\pi }} \int _\mathbb {R}x e^{xu - \frac{x^2}{2}} dx. \end{aligned}$$

This follows from differentiating (2.16) in *u*.

### Proof of Lemma 3.2

Let $G_1$ be as in (3.1) with $\alpha = 1$. Let $-1< t< 0$. From (2.16) and (3.5), we have

$$\begin{aligned} G_1(t, |W_f|^2) W_f&= \frac{1}{(1-t)^2} {{\mathrm{Re\,}}}W_f e^{\frac{-t}{1-t}\big (({{\mathrm{Re\,}}}W_f)^2 + ({{\mathrm{Im\,}}}W_f)^2\big ) }\nonumber \\&\quad \ + \frac{i}{(1-t)^2} {{\mathrm{Im\,}}}W_f e^{\frac{-t}{1-t}\big (({{\mathrm{Re\,}}}W_f)^2 + ({{\mathrm{Im\,}}}W_f)^2\big ) }\nonumber \\&= \frac{1}{\sqrt{-2t}(1-t)^\frac{3}{2}} \frac{1}{2\pi }\int _{\mathbb {R}^2} (x_1+ix_2)\\&\quad \ \times e^{-\frac{x_1^2 + x_2^2}{2}} e^{\sqrt{\frac{-2t}{1-t}}\left( x_1 {{\mathrm{Re\,}}}W_f + x_2 {{\mathrm{Im\,}}}W_f \right) } dx_1 dx_2. \end{aligned}$$

Given $$x_1, x_2, y_1, y_2 \in \mathbb {R}$$, let $$x = x_1 + i x_2$$ and $$y = y_1 + i y_2$$. Then, for any $$ -1< t, s <0$$, from Lemma 2.3, we have

$$\begin{aligned} \int _{\Omega }&G_1(t, W_f(\omega )) W_f(\omega ) \overline{G_1(s, W_h(\omega ))W_h(\omega )} dP(\omega )\nonumber \\ =&\frac{1}{\sqrt{-2t}(1-t)^\frac{3}{2}} \frac{1}{\sqrt{-2s}(1-s)^\frac{3}{2}} \frac{1}{4\pi ^2} \int _{\mathbb {R}^4} x \overline{y}e^{-\frac{|x|^2 + |y|^2 }{2}}\nonumber \\&\times \int _\Omega \exp \Big ( {{\mathrm{Re\,}}}W_{ \sqrt{\frac{-2t}{1-t}}\overline{x} f + \sqrt{\frac{-2s}{1-s}}\overline{y} h}\Big ) dP dx_1dx_2dy_1dy_2 \nonumber \\ =&\frac{1}{\sqrt{-2t}(1-t)^\frac{3}{2}} \frac{1}{\sqrt{-2s}(1-s)^\frac{3}{2}} \frac{1}{4\pi ^2} \int _{\mathbb {R}^4} x\overline{y} e^{-\frac{|x|^2}{2(1-t)}-\frac{|y|^2}{2(1-s)}} \nonumber \\&\times e^{\frac{1}{2} {{\mathrm{Re\,}}}\big ( \sqrt{\frac{-2t}{1-t}} \sqrt{\frac{-2s}{1-s}}\overline{x} y \langle f, h \rangle \big )} dx_1dx_2dy_1dy_2 \nonumber \\ \end{aligned}$$

By a change of variables and applying (2.16) and (3.5), we have

$$\begin{aligned}&= \frac{1}{2 \sqrt{ts}} \frac{1}{4\pi ^2} \int _{\mathbb {R}^4} x\overline{y} e^{-\frac{|x|^2}{2}-\frac{|y|^2}{2}} e^{\sqrt{ts} {{\mathrm{Re\,}}}(\overline{x} y \langle f, h \rangle )} dx_1dx_2dy_1dy_2 \nonumber \\&= \langle f, h \rangle \frac{1}{4\pi } \int _{\mathbb {R}^2} |y|^2 e^{ - \frac{1}{2}(1- ts |\langle f, h \rangle |^2) |y|^2 }dy_1dy_2 \nonumber \\ \end{aligned}$$

By integration by parts, we have

3.6 $$\begin{aligned}&= \frac{\langle f, h \rangle }{1- ts |\langle f, h \rangle |^2} \frac{1}{2\pi } \int _{\mathbb {R}^2} e^{ - \frac{1}{2}(1- ts |\langle f, h \rangle |^2) |y|^2 }dy_1dy_2 \nonumber \\&= \frac{\langle f, h \rangle }{(1- ts |\langle f, h \rangle |^2)^2} = \sum _{k = 0}^\infty (k+1) t^ks^k |\langle f, h \rangle |^{2k}\langle f, h \rangle . \end{aligned}$$

Hence, it follows from (3.1) and (3.6) that

$$\begin{aligned}&\sum _{k = 0}^\infty (k+1) t^ks^k |\langle f, h \rangle |^{2k}\langle f, h \rangle \\&= \sum _{k, m = 0}^\infty t^ks^m \int _\Omega L^{(1)}_k(|W_f(\omega )|^2)W_f \overline{L^{(1)}_m(|W_h(\omega )|^2)W_h} dP(\omega ). \end{aligned}$$

By comparing the coefficients of $t^ks^m$, we obtain (3.4). $\square$

As a preliminary step to the proof of Proposition 1.3, we first estimate the size of the Fourier coefficient of $F_N(u)$.

### Lemma 3.3

Let $m \ge 2$ be an integer. Then, for any $\theta > 0$, there exists $C_{m, \theta } > 0$ such that

3.7 $$\begin{aligned} \Vert \langle F_N(u), e_n \rangle _{L^2_x} \Vert _{L^2(\mu )} \le C_{m, \theta } \frac{1}{(1+ |n|^2)^{\frac{1}{2}(1 - \theta )}} \end{aligned}$$

for any $n \in \mathbb {Z}^2$ and any $N \in \mathbb {N}$. Moreover, given positive $\varepsilon < \frac{1}{2}$ and any $0 < \theta \le 1 - \varepsilon$, there exists $C_{m,\theta , \varepsilon } > 0$ such that

3.8 $$\begin{aligned} \Vert \langle F_M(u) - F_N(u), e_n \rangle _{L^2_x} \Vert _{L^2(\mu )} \le C_{m, \theta , \varepsilon } \frac{1}{N^\varepsilon (1+ |n|^2)^{\frac{1}{2}(1-\theta - \varepsilon )}} \end{aligned}$$

for any $n \in \mathbb {Z}^2$ and any $M \ge N \ge 1$.

### Proof

We first prove (3.7). Let $m \ge 2$ be an integer and $N \in \mathbb {N}$. From (3.3) with (2.23), we have

3.9 $$\begin{aligned} F_N(u) = (-1)^{m+1} (m-1)! \sigma _N^{m-\frac{1}{2} } \cdot \mathbf {P}_N \Big \{ L_{m-1}^{(1)}\big (\big |W_{ \eta _N(x)}\big |^2\big ) \overline{W_{ \eta _N(x)} }\Big \}. \end{aligned}$$

Clearly, $\langle F_N (u), e_n \rangle _{L^2_x} = 0$ when $|n|> N$. Thus, we only need to consider the case $|n|\le N$. From Lemma 3.2 with (3.9), (2.21), and (2.22), we have

3.10 $$\begin{aligned} \Vert \langle F_N (u), e_n \rangle _{L^2_x} \Vert _{L^2(\mu )}^2 =\,&\big [ (m-1)!\big ]^2 \sigma _N^{2m-1} \int _{\mathbb {T}^2_x\times \mathbb {T}^2_y} \overline{e_n(x)} e_n(y) \nonumber \\&\times \int _\Omega L_{m-1}^{(1)}\left( \big |W_{ \eta _N(x)}\big |^2\right) \overline{W_{ \eta _N(x)}} L_{m-1}^{(1)}\big (\big |W_{ \eta _N(y)}\big |^2\big )W_{ \eta _N(y)} dP dx dy\nonumber \\ =\,&m! (m-1)! \int _{\mathbb {T}^2_x\times \mathbb {T}^2_y} |\gamma _N(x-y)|^{2m-2}\gamma _N(x-y) \overline{ e_n(x-y)} dx dy \nonumber \\ =\,&C_m \mathcal {F}\big [ |\gamma _N|^{2m-2}\gamma _N\big ] (n). \end{aligned}$$

Let $\Gamma _{2m-1}(n)$ be as in (2.29). For $(n_1, \dots , n_{2m-1}) \in \Gamma _{2m-1}(n)$, we have $\max _j |n_j| > rsim |n|$. Thus, we have

3.11 $$\begin{aligned} \mathcal {F}\big [ |\gamma _N|^{2m-2}\gamma _N\big ] (n) = \sum _{\begin{array}{c} \Gamma _{2m-1}(n)\\ |n_j| \le N \end{array}} \prod _{j = 1}^{2m-1} \frac{1}{1+ |n_j|^2} \le d_{m, \theta } \frac{1}{(1+|n|^2)^{1-\theta }}. \end{aligned}$$

Hence, (3.7) follows from (3.10) and (3.11).

Next, we prove (3.8). Let $M \ge N \ge 1$. Proceeding as before with (3.9), Lemma 3.2, and (2.22), we have

3.12 $$\begin{aligned}&\Vert \langle F_M(u) - F_N(u), e_n \rangle _{L^2_x} \Vert _{L^2(\mu )}^2\nonumber \\&\quad = C_m \Big \{ \mathbf 1_{[0, M]}(|n|)\mathcal {F}\big [ |\gamma _M|^{2m-2}\gamma _M\big ] (n) - \mathbf 1_{[0, N]}(|n|)\mathcal {F}\big [ |\gamma _N|^{2m-2}\gamma _N\big ] (n) \Big \}\nonumber \\&\quad = C_m \mathbf 1_{[0, N]}(|n|)\Big \{ \mathcal {F}\big [ |\gamma _M|^{2m-2}\gamma _M\big ] (n) - \mathcal {F}\big [ |\gamma _N|^{2m-2}\gamma _N\big ] (n) \Big \}\nonumber \\&\qquad + C_m \mathbf 1_{(N, M]}(|n|)\mathcal {F}\big [ |\gamma _M|^{2m-2}\gamma _M\big ] (n). \end{aligned}$$

On the one hand, noting that $|n| > N$, we can use (3.11) to estimate the second term on the right-hand side of (3.12), yielding (3.8). On the other hand, noting that

$$\begin{aligned}&\Big | \mathcal {F}\big [ |\gamma _M|^{2m-2}\gamma _M\big ] (n) - \mathcal {F}\big [ |\gamma _N|^{2m-2}\gamma _N\big ] (n)\Big | \nonumber \\&\quad \le \sum _{\begin{array}{c} \Gamma _{2m-1}(n)\\ |n_j| \le M\\ \max _j|n_j| \ge N \end{array}} \prod _{j = 1}^{2m-1} \frac{1}{1+|n_j|^2} \le d_{m, \theta } \frac{1}{\max (N^2, 1+|n|^2)^{1-\theta }}, \end{aligned}$$

we can estimate the first term on the right-hand side of (3.12) by (3.8). $\square$

Next, we use the Wiener chaos estimate (Lemma 2.6) to extend Lemma 3.3 for any finite $p\ge 1$.

### Corollary 3.4

Let $m \ge 2$ be an integer. Then, for any $\theta > 0$, there exists $C_{m, \theta } > 0$ such that

3.13 $$\begin{aligned} \Vert \langle F_N(u), e_n \rangle _{L^2_x} \Vert _{L^p(\mu )} \le C_{m, \theta } (p-1)^{m- \frac{1}{2}}\frac{1}{(1+ |n|^2)^{\frac{1}{2} (1-\theta )}} \end{aligned}$$

for any $n \in \mathbb {Z}^2$ and any $N \in \mathbb {N}$. Moreover, given positive $\varepsilon < \frac{1}{2}$ and any $0 < \theta \le 1 - \varepsilon$, there exists $C_{m, \theta , \varepsilon } > 0$ such that

3.14 $$\begin{aligned} \Vert \langle F_M(u) - F_N(u), e_n \rangle _{L^2_x} \Vert _{L^p(\mu )} \le C_{m, \theta , \varepsilon } (p-1)^{m- \frac{1}{2}} \frac{1}{N^\varepsilon (1+ |n|^2)^{\frac{1}{2} (1-\theta -\varepsilon )}} \end{aligned}$$

for any $n \in \mathbb {Z}^2$ and any $M \ge N \ge 1$.

### Proof

Let $m \ge 2$ be an even integer. In view of Lemma 3.3, we only consider the case $p > 2$. From (3.3) with (1.22), we have

$$\begin{aligned} F_N(u) = |u|^{2m - 2}u + \sum _{\ell = 0}^{m-1} a_{m, \ell , N} |u|^{2\ell -2} u. \end{aligned}$$

Recalling (2.29) and (2.30), we have

3.15 $$\begin{aligned} \langle F_N(u), e_n \rangle _{L^2_x} = \sum _{\ell = 0}^{m} a_{m, \ell , N} \sum _{\begin{array}{c} \Gamma _{2\ell -1}(n)\\ |n_j| \le N \end{array}} \prod _{j = 1}^{2\ell -1} \frac{g^*_{n_j}}{\sqrt{1+|n_j|^2}}. \end{aligned}$$

Noting that the right-hand side of (3.15) is a sum of polynomials of degree (at most) $2m-1$ in $\{g_n\}_{n \in \mathbb {Z}^2}$, the bound (3.13) follows from Lemmas 3.3 and 2.6. The proof of (3.14) is analogous and we omit the details. $\square$

Finally, we present the proof of Proposition 1.3.

### Proof of Proposition 1.3

Let $s< 0$. Choose sufficiently small $\theta > 0$ such that $s+ \theta < 0$. Let $p \ge 2$. Then, it follows from Minkowski’s integral inequality and (3.13) that

$$\begin{aligned} \big \Vert \Vert F_N(u) \Vert _{H^s}\big \Vert _{L^p(\mu )}&\le \left( \sum _{n \in \mathbb {Z}^2} \langle n \rangle ^{2s} \Vert \langle F_N(u), e_n \rangle _{L^2_x} \Vert _{L^p(\mu )}^2 \right) ^\frac{1}{2}\\&\lesssim (p-1)^{m- \frac{1}{2}} \left( \sum _{n \in \mathbb {Z}^2} \langle n \rangle ^{-2 +2\theta +2s} \right) ^\frac{1}{2} \le C_{m, p} < \infty \end{aligned}$$

since $s+\theta < 0$. Similarly, given $\varepsilon > 0$ such that $s + \varepsilon < 0$, choose sufficiently small $\theta > 0$ such that $s+ \theta +\varepsilon < 0$. Then, from (3.14), we have

$$\begin{aligned} \big \Vert \Vert F_M(u) - F_N(u) \Vert _{H^s}\big \Vert _{L^p(\mu )}&\le \left( \sum _{n \in \mathbb {Z}^2} \langle n \rangle ^{2s} \Vert \langle F_M(u) - F_N(u), e_n \rangle _{L^2_x} \Vert _{L^p(\mu )}^2 \right) ^\frac{1}{2}\\&\lesssim (p-1)^{m- \frac{1}{2}} \frac{1}{N^\varepsilon } \left( \sum _{n \in \mathbb {Z}^2} \langle n \rangle ^{-2 +2\theta +2\varepsilon + 2s} \right) ^\frac{1}{2}\\&\lesssim (p-1)^{m- \frac{1}{2}} \frac{1}{N^\varepsilon } \end{aligned}$$

since $s + \theta + \varepsilon < 0$. This proves (1.37). $\square$

## Extension to 2-*d* manifolds and domains in $\mathbb {R}^2$

Let $(\mathcal {M},g)$ be a two-dimensional compact Riemannian manifold without boundary or a bounded domain in $\mathbb {R}^2$. In this section, we discuss the extensions of Propositions 1.1, 1.2, and 1.3 to $\mathcal {M}$.

Let $\{\varphi _n\}_{n \in \mathbb {N}}$ be an orthonormal basis of $L^2(\mathcal {M})$ consisting of eigenfunctions of $-\Delta _g$ (with the Dirichlet or Neumann boundary condition when $\mathcal {M}$ is a domain in $\mathbb {R}^2$) with the corresponding eigenvalues $\{\lambda _n^2\}_{n \in \mathbb {N}}$, which we assume to be arranged in the increasing order. Then, by Weyl’s asymptotics, we have

4.1 $$\begin{aligned} \lambda _n\approx n^{\frac{1}{2}}. \end{aligned}$$

See, for example, [46, Chapter 14].

Let $\{g_n(\omega )\}_{n\in \mathbb {N}}$ be a sequence of independent standard complex-valued Gaussian random variables on a probability space $(\Omega , \mathcal {F},P)$. We define the Gaussian measure $\mu$ as the induced probability measure under the map:

4.2 $$\begin{aligned} \omega \in \Omega \longmapsto u(x) = u(x; \omega ) = \sum _{n \in \mathbb {N}}\frac{g_{n}(\omega )}{(1+\lambda ^2_n)^{\frac{1}{2}}}\, \varphi _n(x). \end{aligned}$$

Note that all the results in Sects. 2 and 3 still hold true in this general context with exactly the same proofs, except for Lemmas 2.5 and  3.3, where we used standard Fourier analysis on $\mathbb {T}^2$. In the following, we will instead use classical properties of the spectral functions of the Laplace–Beltrami operator.

Let us now define the Wick renormalization in this context. Let *u* be as in (4.2). Given $N\in \mathbb {N}$, we define the projector $\mathbf {P}_{N}$ by

$$\begin{aligned} u_N = \mathbf {P}_N u= \sum _{\lambda _{n}\le N }\widehat{u}(n) \varphi _{n}. \end{aligned}$$

We also define $\sigma _N$ by

4.3 $$\begin{aligned} \sigma _N (x) = \mathbb {E}[|u_N(x)|^2] = \sum _{\lambda _{n}\le N}\frac{|\varphi _n(x)|^2 }{1+\lambda _{n}^{2}} \lesssim \log N, \end{aligned}$$

where the last inequality follows from [12, Proposition 8.1] and Weyl’s law (4.1). Unlike $\sigma _N$ defined in (1.25) for the flat torus $\mathbb {T}^2$, the function $\sigma _N$ defined above depends on $x \in \mathcal {M}$. Note that $\sigma _N (x) > 0$ for all $x \in \mathcal {M}$. The Wick ordered monomial $:\! |u_N|^{2m} \!:$ is then defined by

4.4 $$\begin{aligned} \, :\! |u_N|^{2m} \!: \, = (-1)^m m! \cdot L_m(|u_N|^2; \sigma _N). \end{aligned}$$

By analogy with (2.19) and (2.20) we define

4.5 $$\begin{aligned} \eta _N(x) (\cdot ):= & {} \frac{1}{\sigma _N^\frac{1}{2}(x)} \sum _{ \lambda _{n} \le N} \frac{\overline{\varphi _n(x)}}{\sqrt{1+{\lambda ^{2}_{n}}}}\varphi _n(\cdot ), \end{aligned}$$

4.6 $$\begin{aligned} \gamma _N (x,y):= & {} \sum _{ \lambda _{n} \le N} \frac{\overline{\varphi _n(x)}\varphi _n(y)}{1+ \lambda _{n}^2}, \end{aligned}$$

for $x, y \in \mathcal {M}$. We simply set $\gamma = \gamma _\infty$ when $N = \infty$.

From the definition (4.3) of $\sigma _{N}$, we have $\Vert \eta _N(x)\Vert _{L^2(\mathcal {M})} = 1$ for all $x \in \mathcal {M}$. Moreover, we have

4.7 $$\begin{aligned} \langle \eta _M(x), \eta _N(y) \rangle _{L^2(\mathcal {M})} = \frac{1}{\sigma _M^\frac{1}{2}(x) \sigma _N^\frac{1}{2}(y)} \gamma _N(x,y) \end{aligned}$$

for all $x,y\in \mathcal {M}$ and $M\ge N$.

We now introduce the spectral function of the Laplace–Beltrami operator on $\mathcal {M}$ as

$$\begin{aligned} \pi _j(x,y)= \sum _{\lambda _n\in (j-1,j]} {\overline{\varphi _n(x)}\varphi _n(y)},\qquad \end{aligned}$$

for $x,y\in \mathcal {M}$ and $j \in \mathbb {Z}_{\ge 0}$. From [40, (1.3) and (1.5) with $q = \infty$], we have the bound $\pi _j(x,x)\le C(j+1)$, uniformly in $x\in \mathcal {M}$. Therefore, by Cauchy–Schwarz inequality, we obtain

4.8 $$\begin{aligned} \vert \pi _j(x,y)\vert \le \sum _{\lambda _n\in (j-1,j]} \vert \varphi _n(x)\vert \vert \varphi _n(y)\vert \le C(j+1), \end{aligned}$$

uniformly in $$x,y\in \mathcal {M}$$.

Let $\sigma$ be a weighted counting measure on $\mathbb {Z}_{\ge 0}$ defined by $\sigma =\sum _{j=0}^{\infty }(j+1)\delta _j$, where $\delta _j$ is the Dirac delta measure at $j \in \mathbb {Z}_{\ge 0}$. We define the operator *L* by

$$\begin{aligned} L: c=\{c_j\}_{j = 0}^\infty \longmapsto \sum _{j=0}^{\infty }c_j \pi _j. \end{aligned}$$

Then, we have the following boundedness of the operator *L*.

### Lemma 4.1

Let $1\le q\le 2$. Then, the operator *L* defined above is continuous from $\ell ^q(\mathbb {Z}_{\ge 0}, \sigma )$ into $L^{q'}(\mathcal {M}^2)$. Here, $q'$ denotes the Hölder conjugate of *q*.

### Proof

By interpolation, it is enough to consider the endpoint cases $q=1$ and $q=2$.

$\bullet$ *Case 1* $q = 1$.    Assume that $c\in \ell ^1(\mathbb {Z}_{\ge 0},\sigma )$. Then, from (4.8), we get

$$\begin{aligned} \vert L(c)(x,y)\vert \le \sum _{j=0}^{\infty }\vert c_j\vert \vert \pi _j(x,y)\vert \le C \sum _{j=0}^{\infty }(j+1)\vert c_j\vert = \Vert c\Vert _{\ell ^1(\sigma )}. \end{aligned}$$

for all $x,y\in \mathcal {M}$. This implies the result for $q=1$.

$\bullet$ *Case 2* $q = 2$.    Assume that $c\in \ell ^2(\mathbb {Z}_{\ge 0},\sigma )$. By the orthogonality of the eigenfunctions $\varphi _n$, we have

4.9 $$\begin{aligned} \int _{\mathcal {M}}\vert L(c)(x,y)\vert ^2 dx =\sum _{j=0}^{\infty }\vert c_j\vert ^2 \pi _j(y,y). \end{aligned}$$

From (4.8) and (4.9), we deduce that

$$\begin{aligned} \int _{\mathcal {M}^2}\vert L(c)(x,y)\vert ^2 dx dy \le C \sum _{j=0}^{\infty }(j+1)\vert c_j\vert ^2 = \Vert c\Vert _{\ell ^2(\sigma )}^2. \end{aligned}$$

This implies the result for $q=2$. $\square$

Next, we extend the definition of $\gamma _N$ to general values of *s*:

$$\begin{aligned} \gamma _{s,N} (x,y) := \sum _{ \lambda _{n} \le N}\frac{\overline{\varphi _n(x)}\varphi _n(y)}{(1+ \lambda ^2_{n})^{\frac{s}{2}}} \end{aligned}$$

for $x,y\in \mathcal {M}$. When $N = \infty$, we simply set $\gamma _s = \gamma _{s, \infty }$ as before. Note that when $s = 2$, $\gamma _{2, N}$ and $\gamma _2$ correspond to $\gamma _N$ and $\gamma$ defined in (4.6).

### Lemma 4.2

Let $s > 1$. Then, the sequence $\{\gamma _{s, N}\}_{N\in \mathbb {N}}$ converges to $\gamma _s$ in $L^p(\mathcal {M}^2)$ for all $2\le p<\frac{2}{2-s}$ when $s < 2$ and $2 \le p \le \infty$ when $s \ge 2$. Moreover, for the same range of *p*, there exist $C>0$ and $\kappa >0$ such that

4.10 $$\begin{aligned} \Vert \gamma _{s, M}-\gamma _{s, N}\Vert _{L^{p}(\mathcal {M}^2)}\le \frac{C}{N^{\kappa }}, \end{aligned}$$

for all $$ M \ge N \ge 1$$.

### Proof

Given $M \ge N \ge 1$, define $\alpha _{N, M}(x, y)$ and $\beta _{N, M}(x, y)$ by

4.11 $$\begin{aligned} \alpha _{N,M}(x,y) :\!&=\gamma _{s, M}(x,y)-\gamma _{s, N}(x,y) \nonumber \\&=\sum _{ N<\lambda _n \le M} \frac{\overline{\varphi _n(x)}\varphi _n(y)}{(1+ \lambda _{n}^2)^\frac{s}{2} }=\sum _{j=N+1}^{M} \sum _{\lambda _n\in (j-1,j]} \frac{\overline{\varphi _n(x)}\varphi _n(y)}{(1+ \lambda _{n}^2)^\frac{s}{2}} \end{aligned}$$

and

$$\begin{aligned} \beta _{N,M}(x,y):\!&=\sum _{j=N+1}^{M} \frac{1}{(1+j^2)^\frac{s}{2}}\sum _{\lambda _n\in (j-1,j]} {\overline{\varphi _n(x)}\varphi _n(y)}= \sum _{j=N+1}^{M} \frac{\pi _j(x,y)}{(1+j^2)^\frac{s}{2}} .\nonumber \end{aligned}$$

Let us first estimate the difference $\alpha _{N,M} - \beta _{N,M}$:

$$\begin{aligned} \vert \alpha _{N,M}(x,y)-\beta _{N,M}(x,y) \vert\le & {} \sum _{j=N+1}^{M} \sum _{\lambda _n\in (j-1,j]} \bigg \vert \frac{1}{(1+ \lambda _{n}^2)^\frac{s}{2}}- \frac{1}{(1+ j^2)^\frac{s}{2}} \bigg \vert \vert \varphi _n(x)\vert \vert \varphi _n(y)\vert \\\le & {} C \sum _{j=N+1}^{M} \frac{1}{j^{s+1}}\sum _{\lambda _n\in (j-1,j]} \vert \varphi _n(x)\vert \vert \varphi _n(y)\vert . \end{aligned}$$

Then, by (4.8), we obtain

4.12 $$\begin{aligned} \vert \alpha _{N,M}(x,y)-\beta _{N,M}(x,y) \vert \le \frac{C}{N^{s-1}}. \end{aligned}$$

Next, we estimate $\beta _{N,M}$. Define a sequence $c = \{ c_j \}_{j = 0}^\infty$ by setting

$$\begin{aligned} c_j= {\left\{ \begin{array}{ll} \frac{1}{(1+j^2)^\frac{s}{2}}, &{}\quad \text {if }N+1\le j \le M,\\ 0, &{} \quad \text {otherwise.} \end{array}\right. } \end{aligned}$$

Note that $c\in \ell ^q(\mathbb {N},\sigma )$ for $\frac{2}{s}<q\le 2$. Hence, it follows from Lemma 4.1 that, given any $2\le p<\frac{2}{2-s}$, there exist $C>0$ and $\kappa > 0$ such that

4.13 $$\begin{aligned} \Vert \beta _{N,M}\Vert _{L^p(\mathcal {M}^2)} =\bigg \Vert \sum _{j=N+1}^{M} \frac{\pi _j}{(1+j^2)^\frac{s}{2}} \bigg \Vert _{L^p(\mathcal {M}^2)} \le C\left( \sum _{j=N+1}^{M} \frac{j+1}{(1+j^2)^{\frac{s}{2}p'}}\right) ^{\frac{1}{p'}} \le \frac{C}{N^\kappa }. \nonumber \\ \end{aligned}$$

The desired estimate (4.10) follows from (4.11), (4.12), and (4.13). $\square$

As in the case of the flat torus, define $G_N$, $N \in \mathbb {N}$, by

$$\begin{aligned} G_N(u) = \frac{1}{2m} \int _{\mathcal {M}}:\! |\mathbf {P}_N u|^{2m}\!: dx. \end{aligned}$$

Then, we have the following extension of Proposition 1.1

### Proposition 4.3

Let $m \ge 2$ be an integer. Then, $\{G_N(u)\}_{N\in \mathbb {N}}$ is a Cauchy sequence in $L^p( \mu )$ for any $p\ge 1$. More precisely, there exists $C_{m} > 0$ such that

4.14 $$\begin{aligned} \Vert G_M(u) - G_N(u) \Vert _{L^p(\mu )} \le C_{m} (p-1)^m\frac{1}{N^\frac{1}{2}} \end{aligned}$$

for any $p \ge 1$ and any $M \ge N \ge 1$.

As in Sect. 2, we make use of the white noise functional on $L^2(\mathcal {M})$. Let $w(x; \omega )$ be the mean-zero complex-valued Gaussian white noise on $\mathcal {M}$ defined by

$$\begin{aligned} w(x; \omega ) = \sum _{n \in \mathbb {N}} g_n(\omega ) \varphi (x). \end{aligned}$$

We then define the white noise functional $W_{(\cdot )}: L^2(\mathcal {M}) \rightarrow L^2(\Omega )$ by

4.15 $$\begin{aligned} W_f = \langle f, w(\omega ) \rangle _{L^2(\mathcal {M})} = \sum _{n \in \mathbb {N}} \widehat{f}(n) \overline{g_n(\omega )}. \end{aligned}$$

Note that Lemma 2.3 and hence Lemma 2.4 also hold on $\mathcal {M}$.

### Proof

Thanks to the Wiener chaos estimate (Lemma 2.6), we are reduced to the case $p=2$. Given $N \in \mathbb {N}$ and $x \in \mathbb {T}^{2}$, it follows from (4.3), (4.5), and (4.15) that

4.16 $$\begin{aligned} u_N (x) = \sigma _N^\frac{1}{2}(x) \frac{u_N(x)}{\sigma _N^\frac{1}{2}(x) } = \sigma _N^{\frac{1}{2}}(x) \overline{W_{ {\eta _N(x)}}}. \end{aligned}$$

Then, from (4.4) and (4.16), we have

4.17 $$\begin{aligned} :\! |u_N|^{2m} \!: \, = (-1)^m m! \sigma ^m_NL_m\bigg (\frac{| u_N|^2}{\sigma _N}\bigg ) \, = (-1)^m m! \sigma _N^m L_m\big (\big |W_{{\eta _N(x)}}\big |^2\big ). \end{aligned}$$

Hence, from (4.17), Lemma 2.4, and (4.7), we have

$$\begin{aligned} (2m)^{2} \Vert&G_M(u) - G_N(u) \Vert _{L^2(\mu )}^2\\&= (m!)^2 \int _{\mathcal {M}_x\times \mathcal {M}_y} \int _{\Omega } \Big [ \sigma _M^{m}(x) \sigma _M^{m} (y)L_m\left( \big |W_{{\eta _M(x)}}\big |^2\right) L_m\left( \big |W_{{\eta _M(y)}}\big |^2\right) \nonumber \\&\quad - \sigma _M^{m}(x)\sigma _N^{m} (y)L_m\left( \big |W_{{\eta _M(x)}}\big |^2\right) L_m\left( \big |W_{{\eta _N(y)}}\big |^2\right) \nonumber \\&\quad - \sigma _N^{m}(x)\sigma _M^{m} (y)L_m\left( \big |W_{{\eta _N(x)}}\big |^2\right) L_m\left( \big |W_{{\eta _M(y)}}\big |^2\right) \nonumber \\&\quad + \sigma _N^{m}(x)\sigma _N^{m}(y) L_m\left( \big |W_{{\eta _N(x)}}\big |^2\right) L_m\left( \big |W_{{\eta _N(y)}}\big |^2\right) \Big ] dP dx dy \nonumber \\&= (m!)^2 \int _{\mathcal {M}_x\times \mathcal {M}_y} \big [ |\gamma _M(x,y)|^{2m} - |\gamma _N(x,y)|^{2m}\big ] dx dy. \end{aligned}$$

The desired estimate (4.14) for $p = 2$ follows from Hölder’s inequality and Lemma 4.2. $\square$

### Remark 4.4

Observe that the renormalization procedure (4.4) uses less spectral information than the one used in [12, Section 8] for the case $m=2$. Namely, the approach in [12] needed an explicit expansion of the spectral function (see [12, Proposition 8.7]), but the inequality (4.8) is enough in the argument above.

The function $\gamma$ defined in (4.6) is the Green function of the operator $1-\Delta$. It is well-known (see for example Aubin [2, Theorem 4.17]) that it enjoys the bound

4.18 $$\begin{aligned} |\gamma (x,y)|\le C\big |\log (d(x,y))\big |, \end{aligned}$$

where *d*(*x*, *y*) is the distance on $\mathcal {M}$ between the points $x,y\in \mathcal {M}$. The bound (4.18) implies that $\gamma \in L^{p}(\mathcal {M}^{2})$ for all $1\le p<\infty$. However, we do not know whether $\gamma _{N}$ (which is the Green function of a spectral truncation of $1-\Delta$) satisfies a similar bound, uniformly in *N*. This could have given an alternative proof. We refer to [12, Remark 8.4] for a discussion on these topics.

All the definitions and notations from (1.28) to (1.36) have obvious analogues in the general case of the manifold $\mathcal {M}$, and thus we do not redefine them here.

For $$N \in \mathbb {N}$$, let

$$\begin{aligned} R_N(u) = e^{-G_{N}(u)} = e^{-\frac{1}{2m} \int _{\mathcal {M}} : |u_N|^{2m} : \, dx}. \end{aligned}$$

In view of (4.3) and (4.17), the logarithmic upper bound (2.31) on $-G_N(u)$ also holds on the manifold $\mathcal {M}$. Hence, by proceeding as in the case of the flat torus, we have the following analogue of Proposition 1.2.

### Proposition 4.5

Let $m \ge 2$ be an integer. Then, $R_N(u) \in L^p(\mu )$ for any $p\ge 1$ with a uniform bound in *N*, depending on $p \ge 1$. Moreover, for any finite $p \ge 1$, $R_N(u)$ converges to some *R*(*u*) in $L^p(\mu )$ as $N \rightarrow \infty$.

We conclude this section by the following analogue of Proposition 1.3, which enables us to define the Wick ordered nonlinearity $:\! |u|^{2(m-1)} u \!:$ on the manifold $\mathcal {M}$.

### Proposition 4.6

Let $$m \ge 2$$ be an integer and $$ s< 0$$. Then, $$\{F_N(u)\}_{N\in \mathbb {N}}$$ defined in (1.36) and (3.3) is a Cauchy sequence in $$L^p(\mu ; H^s(\mathcal {M}))$$ for any $$p\ge 1$$. More precisely, there exist $$\kappa >0 $$ and $$C_{m, s, \kappa } > 0$$ such that

4.19 $$\begin{aligned} \big \Vert \Vert F_M(u) - F_N(u) \Vert _{H^s}\big \Vert _{L^p(\mu )} \le C_{m, s, \kappa } (p-1)^{m-\frac{1}{2}}\frac{1}{N^\kappa } \end{aligned}$$

for any $p \ge 1$ and any $M \ge N \ge 1$.

### Proof

Given $N, n \in \mathbb {N}$, define $J_{N, n}$ by

$$\begin{aligned} J_{N,n}=m! (m-1)! \int _{\mathcal {M}_x\times \mathcal {M}_y} |\gamma _{2,N}(x,y)|^{2m-2}\overline{\gamma _{2,N}(x,y)}\overline{\varphi _{n}(x)}\varphi _{n}(y) dx dy . \end{aligned}$$

Then, proceeding as in (3.10) and (3.12) with (3.9), Lemma 3.2, and (4.7), we obtain

$$\begin{aligned} \Vert \langle F_M(u) - F_N(u), \varphi _n \rangle _{L^2_x} \Vert _{L^2(\mu )}^2 = \mathbf 1_{[0, N]}(\lambda _{n})\big ( J_{M,n}-J_{N,n}\big ) + \mathbf 1_{(N, M]}(\lambda _{n})J_{M,n} \end{aligned}$$

for $M \ge N \ge 1$. With $\varepsilon =-s>0$, we then obtain

$$\begin{aligned}&\big \Vert \Vert F_M(u) - F_N(u) \Vert _{H^{-\varepsilon }}\big \Vert ^2_{L^2(\mu )} = \sum _{n\ge 1} \frac{1}{(1+\lambda _n^{2})^{\varepsilon }} \Vert \langle F_M(u) - F_N(u), \varphi _n \rangle _{L^2_x}\Vert ^2_{L^2(\mu )} \\&\quad =\sum _{\lambda _n \le N} \frac{1}{(1+\lambda _n^{2})^{\varepsilon }} (J_{M,n}-J_{N,n}) +\sum _{N<\lambda _n \le M} \frac{1}{(1+\lambda _n^{2})^{\varepsilon }} J_{M,n} \\&\quad = C_m \int _{\mathcal {M}_x\times \mathcal {M}_y} \big ( |\gamma _{2,M}|^{2m-2}\overline{\gamma _{2,M}} - |\gamma _{2,N}|^{2m-2}\overline{\gamma _{2,N}} \big )\gamma _{2\varepsilon ,N}(x,y) dx dy\\&\qquad + C_m \int _{\mathcal {M}_x\times \mathcal {M}_y} |\gamma _{2,M}|^{2m-2}\overline{\gamma _{2,M}} \big (\gamma _{2\varepsilon ,M} -\gamma _{2\varepsilon ,N}\big )(x,y)dx dy\\&\quad =:A_{N,M}+B_{N,M}. \end{aligned}$$

In the following, We only bound the term $B_{N,M}$, since the first term $A_{N,M}$ can be handled similarly. Set $\langle \nabla _x \rangle =(1-\Delta _x)^{\frac{1}{2}}$. Then, noting that $\langle \nabla _x \rangle ^{-1+\varepsilon } \gamma _{2\varepsilon }=\gamma _{1+\varepsilon }$ and that ${\langle \nabla _x \rangle ^{1-\varepsilon } \gamma _{2}=\gamma _{1+\varepsilon }}$, it follows from Cauchy–Schwarz inequality and the fractional Leibniz rule that

$$\begin{aligned} B_{N,M}&= C_m \int _{\mathcal {M}_x \times \mathcal {M}_y} \langle \nabla _x \rangle ^{1-\varepsilon }\big ( |\gamma _{2,M}|^{2m-2}\overline{\gamma _{2,M}}(x,y) \big )\langle \nabla _x \rangle ^{-1+\varepsilon }( \gamma _{2\varepsilon ,M} -\gamma _{2\varepsilon ,N}) (x,y)dx dy\\&= C_m \int _{\mathcal {M}_x \times \mathcal {M}_y} \langle \nabla _x \rangle ^{1-\varepsilon }\big ( |\gamma _{2,M}|^{2m-2}\overline{\gamma _{2,M}}(x,y) \big )( \gamma _{1+\varepsilon ,M} -\gamma _{1+\varepsilon ,N}) (x,y)dx dy.\\&\le C_m \big \Vert \langle \nabla _x \rangle ^{1-\varepsilon }\big ( |\gamma _{2,M}|^{2m-2}\overline{\gamma _{2,M}} \big ) \big \Vert _{L^2(\mathcal {M}^2)} \Vert \gamma _{1+\varepsilon ,M} -\gamma _{1+\varepsilon ,N} \Vert _{L^2(\mathcal {M}^2)} \\&\lesssim \big \Vert \gamma _{1+\varepsilon ,M} \big \Vert _{L^{p_\varepsilon }(\mathcal {M}^2)} \big \Vert \gamma _{2,M} \big \Vert ^{2m-2}_{L^{q_\varepsilon }(\mathcal {M}^2)} \Vert \gamma _{1+\varepsilon ,M} -\gamma _{1+\varepsilon ,N} \Vert _{L^2(\mathcal {M}^2)} \end{aligned}$$

with $p_{\varepsilon }=\frac{2}{1-\varepsilon /2}$ and $q_{\varepsilon }=8(m-1)/\varepsilon$. Hence, from Lemma 4.2 we conclude that

$$\begin{aligned} B_{N,M} \le \frac{C_{m, \varepsilon }}{N^\kappa }.\end{aligned}$$

By estimating $A_{N, M}$ in an analogous manner, we obtain

4.20 $$\begin{aligned} \big \Vert \Vert F_M(u) - F_N(u) \Vert _{H^{-\varepsilon }}\big \Vert _{L^2(\mu )} \le \frac{C_{m, \varepsilon }}{N^\kappa }. \end{aligned}$$

The bound (4.19) for general $p\ge 2$ follows from (4.20) and the Wiener chaos estimate (Lemma 2.6). $\square$

## Proof of Theorems 1.4 and  1.5

In this section, we present the proof of Theorem 1.5 on a manifold $\mathcal {M}$ (which contains a particular case of the flat torus stated in Theorem 1.4). Fix an integer $m \ge 2$ and $s < 0$ in the remaining part of this section. We divide the proof into three subsections. In Sect. 5.1, we first construct global-in-time dynamics for the truncated Wick ordered NLS and prove that the corresponding truncated Gibbs measures $P^{(2m)}_{2, N}$ are invariant under its dynamics. Then, we construct a sequence $\{\nu _N\}_{N\in \mathbb {N}}$ of probability measures on space-time functions such that their marginal distributions at time *t* are precisely given by the truncated Gibbs measures $P^{(2m)}_{2, N}$. In Sect. 5.2, we prove a compactness property of $\{\nu _N\}_{N\in \mathbb {N}}$ so that $\nu _N$ converges weakly up to a subsequence. In Sect. 5.3, by Skorokhod’s theorem (Lemma 5.7), we upgrade this weak convergence of $\nu _N$ to almost sure convergence of new $C(\mathbb {R}; H^s)$-valued random variables, whose laws are given by $\nu _N$, and complete the proof of Theorem 1.5.

### Extending the truncated Gibbs measures onto space-time functions

Recall that $\mathbf {P}_{N}$ is the spectral projector onto the frequencies $\big \{n \in \mathbb {N}: \lambda _{n}\le N\big \}$. Consider the truncated Wick ordered NLS:

5.1 $$\begin{aligned} i \partial _tu^N + \Delta u^N = \mathbf {P}_N\big (\! :\!|\mathbf {P}_Nu^N|^{2(m-1)} \mathbf {P}_Nu^N\!: \!\big ). \end{aligned}$$

We first prove global well-posedness of (5.1) and invariance of the truncated Gibbs measure $P^{(2m)}_{2, N}$ defined in  (1.30):

$$\begin{aligned} d P^{(2m)}_{2, N}= Z_N^{-1} R_N(u) d\mu = Z_N^{-1} e^{-\frac{1}{2m} \int _{\mathcal {M}} :| u_N|^{2m} : \, dx} d\mu . \end{aligned}$$

#### Lemma 5.1

Let $N \in \mathbb {N}$. Then, the truncated Wick ordered NLS (5.1) is globally well-posed in $H^s(\mathcal {M})$. Moreover, the truncated Gibbs measure $P^{(2m)}_{2, N}$ is invariant under the dynamics of (5.1).

#### Proof

We first prove global well-posedness of the truncated Wick ordered NLS (5.1). Given $N \in \mathbb {N}$, let $v^N = \mathbf {P}_N u^N$. Then, (5.1) can be decomposed into the nonlinear evolution equation for $v^N$ on the low frequency part $\{\lambda _{n}\le N\}$:

5.2 $$\begin{aligned} i \partial _tv^N + \Delta v^N = \mathbf {P}_N\big (\! :\!|v^N|^{2(m-1)} v^N\!: \!\big ) \end{aligned}$$

and a linear ODE for each high frequency $\lambda _{n} > N$:

5.3 $$\begin{aligned} i \partial _t\widehat{u^N}(n) = \lambda _{n}^2 \widehat{u^N}(n). \end{aligned}$$

As a linear equation, any solution $\widehat{u^N}(n)$ to (5.3) exists globally in time. By viewing (5.2) on the Fourier side, we see that (5.2) is a finite dimensional system of ODEs of dimension $d_{N}=\#\big \{n\;:\, \lambda _{n}\le N\big \}$, where the vector field depends smoothly on $\big \{\widehat{u^N}(n)\big \}_{\lambda _{n}\le N}$. Hence, by the Cauchy–Lipschitz theorem, we obtain local well-posedness of (5.2).

With (3.3) we have

$$\begin{aligned} \frac{d}{dt}\int _{\mathcal {M}} |v^N|^2 dx&= 2{{\mathrm{Re\,}}}\int _{\mathcal {M}} \partial _tv^N \overline{v^N} dx\\&= - 2{{\mathrm{Re\,}}}\left( i \int _{\mathcal {M}} |\nabla v^N|^2 dx\right) \\&\quad - 2 (-1)^{m+1} (m-1)! \sigma _N^{m-1} {{\mathrm{Re\,}}}\left( i \int _{\mathcal {M}} L_{m-1}^{(1)}\left( \tfrac{|v^N|^2}{\sigma _N}\right) |v^N|^2dx \right) \\&= 0. \end{aligned}$$

In particular, this shows that the Euclidean norm

$$\begin{aligned} \big \Vert \{\widehat{v^N}(n)\}_{\lambda _{n}\le N}\big \Vert _{\mathbb {C}^{d_{N}}} = \left( \sum _{\lambda _{n} \le N} |\widehat{v^N}(n)|^2\right) ^\frac{1}{2} = \left( \int _{\mathcal {M}} |v^N|^2 dx \right) ^\frac{1}{2} \end{aligned}$$

is conserved under (5.2). This proves global existence for (5.2) and hence for the truncated Wick ordered NLS (5.1).

As in (1.34), write $P^{(2m)}_{2, N}= \widehat{P}^{(2m)}_{2, N}\otimes \mu ^\perp _N$. On the one hand, the Gaussian measure $\mu _N^\perp$ on the high frequencies $\{\lambda _{n} > N\}$ is clearly invariant under the linear flow (5.3). On the other hand, noting that (5.2) is the finite dimensional Hamiltonian dynamics corresponding to $H^N_{\mathrm{Wick}}(v^N)$ with

$$\begin{aligned} H^N_{\mathrm{Wick}}(v^N) = \frac{1}{2}\int _{\mathcal {M}} |\nabla v^N|^2dx + \frac{1}{2m} \int _{\mathcal {M}} :\! | v^N|^{2m}\!: dx, \end{aligned}$$

we see that $\widehat{P}^{(2m)}_{2, N}$ is invariant under (5.2). Therefore, the truncated Gibbs measure $P^{(2m)}_{2, N}$ is invariant under the dynamics of (5.1). $\square$

Let $\Phi _N: H^s (\mathcal {M})\rightarrow C(\mathbb {R}; H^s(\mathcal {M}))$ be the solution map to (5.1) constructed in Lemma 5.1. For $t \in \mathbb {R}$, we use $\Phi _N(t): H^s(\mathcal {M}) \rightarrow H^s(\mathcal {M})$ to denote the map defined by $\Phi _N(t)(\phi ) = \big (\Phi _N(\phi )\big )(t)$. We endow $C(\mathbb {R}; H^s(\mathcal {M}))$ with the compact-open topology. Namely, we can view $C(\mathbb {R}; H^s(\mathcal {M}))$ as a Fréchet space endowed with the following metric:

$$\begin{aligned} d(u, v) = \sum _{j = 1}^\infty \frac{1}{2^j} \frac{\Vert u-v\Vert _{ C([-j, j]; H^s)}}{1+\Vert u-v\Vert _{ C([-j, j]; H^s)}}.\end{aligned}$$

Under this topology, a sequence $\{u_n\}_{n \in \mathbb {N}}\subset C(\mathbb {R}; H^s(\mathcal {M}))$ converges if and only if it converges uniformly on any compact time interval. Then, it follows from the local Lipschitz continuity of $\Phi _N(\cdot )$ that $\Phi _N$ is continuous from $H^s (\mathcal {M})$ into $C(\mathbb {R}; H^s(\mathcal {M}))$. We now extend $P^{(2m)}_{2, N}$ on $H^s$ to a probability measure $\nu _N$ on $C(\mathbb {R}; H^s(\mathcal {M}))$ by setting

$$\begin{aligned} \nu _N = P^{(2m)}_{2, N}\circ \Phi _N^{-1}. \end{aligned}$$

Namely, $\nu _N$ is the induced probability measure of $P^{(2m)}_{2, N}$ under the map $\Phi _N$. In particular, we have

5.4 $$\begin{aligned} \int _{C(\mathbb {R}; H^s)} F(u) d\nu _N (u) = \int _{H^s} F(\Phi _N(\phi )) d P^{(2m)}_{2, N}(\phi ) \end{aligned}$$

for any measurable function $F :C(\mathbb {R}; H^s(\mathcal {M}))\rightarrow \mathbb {R}$.

### Tightness of the measures $\nu _N$

In the following, we prove that the sequence $\{\nu _N\}_{N\in \mathbb {N}}$ of probability measures on $C(\mathbb {R}; H^s(\mathcal {M}))$ is precompact. Recall the following definition of tightness of a sequence of probability measures.

#### Definition 5.2

A sequence $\{ \rho _n\}_{n \in \mathbb {N}}$ of probability measures on a metric space $\mathcal {S}$ is *tight* if, for every $\varepsilon > 0$, there exists a compact set $K_\varepsilon$ such that $\rho _n(K_\varepsilon ^c) \le \varepsilon$ for all $n \in \mathbb {N}$.

It is well known that tightness of a sequence of probability measures is equivalent to precompactness of the sequence. See [3].

#### Lemma 5.3

(Prokhorov’s theorem) If a sequence of probability measures on a metric space $\mathcal {S}$ is tight, then there is a subsequence that converges weakly to a probability measure on $\mathcal {S}$.

The following proposition shows that the family $\{ \nu _N\}_{N \in \mathbb {N}}$ is tight and hence, up to a subsequence, it converges weakly to some probability measure $\nu$ on $C(\mathbb {R}; H^s)$.

#### Proposition 5.4

Let $s< 0$. Then, the family $\{ \nu _N\}_{N \in \mathbb {N}}$ of the probability measures on $C(\mathbb {R}; H^s(\mathcal {M}))$ is tight.

The proof of Proposition 5.4 is similar to that of [12, Proposition 4.11]. While [12, Proposition 4.11] proves the tightness of $\{ \nu _N\}_{N \in \mathbb {N}}$ restricted to $[-T, T]$ for each $T>0$, we directly prove the tightness of $\{ \nu _N\}_{N \in \mathbb {N}}$ on the whole time interval.

In the following, we first state several lemmas. We present the proof of Proposition 5.4 at the end of this subsection. For simplicity of presentation, we use the following notations. Given $T>0$, we write $L^p_T H^s$ for $L^p([-T, T]; H^s)$. We use a similar abbreviation for other function spaces in time. Let $\rho$ be a probability measure on $H^s$. With a slight abuse of notation, we use $L^p(\rho )H^s$ to denote

$$\begin{aligned} \Vert \phi \Vert _{L^p(\rho )H^s} = \big \Vert \Vert \phi \Vert _{H^s} \big \Vert _{L^p(\rho )}. \end{aligned}$$

The first lemma provides a control on the size of random space-time functions. The invariance of $P^{(2m)}_{2, N}$ under the dynamics of (5.1) plays an important role.

#### Lemma 5.5

Let $$ s< 0$$ and $$p \ge 1$$. Then, there exists $$C_p > 0$$ such that

5.5 $$\begin{aligned} \big \Vert \Vert u\Vert _{L^p_T H^s} \big \Vert _{L^p(\nu _N)}&\le C_p T^\frac{1}{p}, \end{aligned}$$

5.6 $$\begin{aligned} \big \Vert \Vert u\Vert _{W^{1, p}_T H^{s-2}} \big \Vert _{L^p(\nu _N)}&\le C_pT^\frac{1}{p}, \end{aligned}$$

uniformly in $$N \in \mathbb {N}$$.

#### Proof

By Fubini’s theorem, the definition  (5.4), the invariance of $P^{(2m)}_{2, N}$ (Lemma 5.1), and Hölder’s inequality, we have

5.7 $$\begin{aligned} \big \Vert \Vert u\Vert _{L^p_T H^s} \big \Vert _{L^p(\nu _N)}&= \big \Vert \Vert \Phi _N(t) (\phi ) \Vert _{L^p_T H^s} \big \Vert _{L^p(P^{(2m)}_{2, N})} = \big \Vert \Vert \Phi _N(t) (\phi ) \Vert _{L^p(P^{(2m)}_{2, N}) H^s} \big \Vert _{L^p_T} \nonumber \\&= (2T)^\frac{1}{p} \Vert \phi \Vert _{L^p(P^{(2m)}_{2, N}) H^s} \le (2T)^\frac{1}{p}\Vert R_N \Vert _{L^{2p}(\mu )} \Vert \phi \Vert _{L^{2p}(\mu ) H^s}. \end{aligned}$$

Then, (5.5) follows from (5.7) with Proposition 4.5, (4.2), and Lemma 2.6.

From (5.1) and the definition of $F_{N}$, we have

5.8 $$\begin{aligned} \big \Vert \Vert \partial _tu\Vert _{L^p_T H^{s-2}} \big \Vert _{L^p(\nu _N)} \le \big \Vert \Vert u\Vert _{L^p_T H^{s}} \big \Vert _{L^p(\nu _N)} + \big \Vert \Vert F_N(u)\Vert _{L^p_T H^{s-2}} \big \Vert _{L^p(\nu _N)} . \end{aligned}$$

The first term is estimated by (5.5). Proceeding as in (5.7) with Propositions 4.5 and 4.6, we have

$$\begin{aligned} \big \Vert \Vert F_N(u)\Vert _{L^p_T H^{s-2}} \big \Vert _{L^p(\nu _N)}&\le (2T)^\frac{1}{p}\Vert R_N \Vert _{L^{2p}(\mu )} \Vert F_N(\phi ) \Vert _{L^{2p}(\mu ) H^{s-1}} \le C_p T^\frac{1}{p}. \end{aligned}$$

This proves (5.6). $$\square $$

Recall the following lemma on deterministic functions from [12].

#### Lemma 5.6

([12, Lemma 3.3]) Let $T > 0$ and $1\le p \le \infty$. Suppose that $u \in L^p_T H^{s_1}$ and $\partial _tu \in L^p_T H^{s_2}$ for some $s_2 \le s_1$. Then, for $\delta > p^{-1}(s_1 - s_2)$, we have

$$\begin{aligned} \Vert u \Vert _{L^\infty _TH^{s_1 - \delta }} \lesssim \Vert u \Vert _{L^p_T H^{s_1}}^{1-\frac{1}{p}} \Vert u \Vert _{W^{1, p}_T H^{s_2}}^{ \frac{1}{p}}.\end{aligned}$$

Moreover, there exist $\alpha > 0$ and $\theta \in [0, 1]$ such that for all $t_1, t_2 \in [-T, T]$, we have

$$\begin{aligned} \Vert u(t_2) - u(t_1) \Vert _{H^{s_1 - 2\delta }} \lesssim |t_2 - t_1|^\alpha \Vert u \Vert _{L^p_T H^{s_1}}^{1-\theta } \Vert u \Vert _{W^{1, p}_T H^{s_2}}^{ \theta }.\end{aligned}$$

We are now ready to present the proof of Proposition 5.4.

#### Proof of Proposition 5.4

Let $s< s_1< s_2 < 0$. For $\alpha \in (0, 1)$, consider the Lipschitz space $C^\alpha _TH^{s_1} = C^\alpha ([-T, T]; H^{s_1}(\mathcal {M}))$ defined by the norm

$$\begin{aligned} \Vert u \Vert _{C^\alpha _T H^{s_1}} = \sup _{\begin{array}{c} t_1, t_2 \in [-T, T]\\ t_1 \ne t_2 \end{array}} \frac{\Vert u(t_1) - u(t_2) \Vert _{H^{s_1}}}{|t_1 - t_2|^\alpha } + \Vert u \Vert _{L^\infty _T H^{s_1}}. \end{aligned}$$

It follows from the Arzelà-Ascoli theorem that the embedding $C^\alpha _T H^{s_1} \subset C_T H^{s}$ is compact for each $T>0$.

By Lemma 5.6 with large $p\gg 1$ and Young’s inequality, we have

5.9 $$\begin{aligned} \Vert u \Vert _{C^\alpha _T H^{s_1}} \lesssim \Vert u \Vert _{L^p_TH^{s_2}}^{1-\theta } \Vert u \Vert _{W^{1, p}_TH^{s_2 - 2}}^{\theta } \lesssim \Vert u \Vert _{L^p_TH^{s_2}}+ \Vert u \Vert _{W^{1, p}_TH^{s_2 - 2}} \end{aligned}$$

for some $\alpha \in (0, 1)$. Then, it follows from (5.9) and Lemma 5.5 that

5.10 $$\begin{aligned} \big \Vert \Vert u \Vert _{C^\alpha _T H^{s_1}}\big \Vert _{L^p(\nu _N)} \le C_p T^\frac{1}{p}. \end{aligned}$$

For $j \in \mathbb {N}$, let $T_j = 2^j$. Given $\varepsilon > 0$, define $K_\varepsilon$ by

$$\begin{aligned} K_\varepsilon = \big \{ u \in C(\mathbb {R}; H^s):\, \Vert u \Vert _{C^\alpha _{T_j} H^{s_1}} \le c_0 \varepsilon ^{-1} T_j^{1+ \frac{1}{p}} \quad \text { for all } j \in \mathbb {N}\big \}.\end{aligned}$$

Then, by Markov’s inequality with (5.10) and choosing $c_0 > 0$ sufficiently large, we have

$$\begin{aligned} \nu _N(K_\varepsilon ^c) \le c^{-1}_0 C_1 \varepsilon T_j^{-1-\frac{1}{p}} \big \Vert \Vert u \Vert _{C^\alpha _{T_j} H^{s_1}}\big \Vert _{L^p(\nu _N)} \le c^{-1}_0 C_p \varepsilon \sum _{j = 1}^\infty T_j^{-1} = c^{-1}_0 C_p \varepsilon < \varepsilon .\end{aligned}$$

Hence, it remains to prove that $K_\varepsilon$ is compact in $C(\mathbb {R}; H^s)$ endowed with the compact-open topology. Let $\{u_n \}_{n \in \mathbb {N}} \subset K_\varepsilon$. By the definition of $K_\varepsilon$, $\{u_n \}_{n \in \mathbb {N}}$ is bounded in $C^\alpha _{T_j} H^{s_1}$ for each $j \in \mathbb {N}$. Then, by a diagonal argument, we can extract a subsequence $\{u_{n_\ell } \}_{\ell \in \mathbb {N}}$ convergent in $C^\alpha _{T_j} H^s$ for each $j \in \mathbb {N}$. In particular, $\{u_{n_\ell } \}_{\ell \in \mathbb {N}}$ converges uniformly in $H^s$ on any compact time interval. Hence, $\{u_{n_\ell } \}_{\ell \in \mathbb {N}}$ converges in $C(\mathbb {R}; H^s)$ endowed with the compact-open topology. This proves that $K_\varepsilon$ is compact in $C(\mathbb {R}; H^s)$. $\square$

### Proof of Theorem 1.5

It follows from Proposition 5.4 and Lemma 5.3 that, passing to a subsequence, $\nu _{N_j}$ converges weakly to some probability measure $\nu$ on $C(\mathbb {R}; H^s(\mathcal {M}))$ for any $s< 0$. The following Skorokhod’s theorem tells us that, by introducing a new probability space $(\widetilde{\Omega }, \mathcal {F}, \widetilde{P})$ and a sequence of new random variables $\widetilde{u^N}$ with the same distribution $\nu _N$, we can upgrade this weak convergence to almost sure convergence of $\widetilde{u^N}$. See [3].

#### Lemma 5.7

(Skorokhod’s theorem) Let $\mathcal {S}$ be a complete separable metric space. Suppose that $\rho _n$ are probability measures on $\mathcal {S}$ converging weakly to a probability measure $\rho$. Then, there exist random variables $X_n:\widetilde{\Omega } \rightarrow \mathcal {S}$ with laws $\rho _n$ and a random variable $X:\widetilde{\Omega } \rightarrow \mathcal {S}$ with law $\rho$ such that $X_n \rightarrow X$ almost surely.

By Lemma 5.7, there exist another probability space $(\widetilde{\Omega }, \widetilde{\mathcal {F}}, \widetilde{P})$, a sequence $\big \{ \widetilde{u^{N_j}}\big \}_{j \in \mathbb {N}}$ of $C(\mathbb {R}; H^s)$-valued random variables, and a $C(\mathbb {R}; H^s)$-valued random variable *u* such that

5.11 $$\begin{aligned} \mathcal {L}\big (\widetilde{u^{N_j}}\big ) = \mathcal {L}( u^{N_j}) = \nu _N, \qquad \mathcal {L}(u) = \nu , \end{aligned}$$

and $\widetilde{u^{N_j}}$ converges to *u* in $C(\mathbb {R}; H^s)$ almost surely with respect to $\widetilde{P}$.

Next, we determine the distributions of these random variables at a given time *t*. By Lemma 5.1, we have

5.12 $$\begin{aligned} \mathcal {L}( u^{N_j}(t)) = P^{(2m)}_{2, N_j} \end{aligned}$$

for each $$t \in \mathbb {R}$$.

#### Lemma 5.8

Let $\widetilde{u}_{N_j}$ and *u* be as above. Then, we have

$$\begin{aligned} \mathcal {L}\big (\widetilde{u^{N_j}}(t)\big ) = P^{(2m)}_{2, N_j} \quad \text {and} \quad \mathcal {L}(u(t)) = P^{(2m)}_2\end{aligned}$$

for any $$t \in \mathbb {R}$$.

#### Proof

Fix $t \in \mathbb {R}$. Let $R_t:C(\mathbb {R}; H^s) \rightarrow H^s$ be the evaluation map defined by $R_t(v) = v(t)$. Note that $R_t$ is continuous. From (5.12), we have

5.13 $$\begin{aligned} P^{(2m)}_{2, N_j} = \nu _{N_j}\circ R_t^{-1}. \end{aligned}$$

Then, it follows from (5.11) and (5.13) that

5.14 $$\begin{aligned} \mathcal {L}\big (\widetilde{u^{N_j}}(t)\big ) = \nu _{N_j}\circ R_t^{-1} = P^{(2m)}_{2, N_j} . \end{aligned}$$

On the one hand, since $$\widetilde{u^{N_j}}$$ converges to *u* in $$C(\mathbb {R}; H^s)$$ almost surely with respect to $$\widetilde{P}$$, $$\widetilde{u^{N_j}}(t)$$ converges to *u*(*t*) in $$H^s$$ almost surely. As a consequence, we see that $$\mathcal {L}\big (\widetilde{u^{N_j}}(t)\big )$$ converges weakly to $$ \mathcal {L}\big ({u}(t)\big ) $$. On the other hand, it follows from Proposition 4.5 that $$\mathcal {L}\big (\widetilde{u^{N_j}}(t)\big )= P^{(2m)}_{2, N_j}$$ converges to $$ P^{(2m)}_{2}$$. Therefore, from the uniqueness of the limit, we conclude that $$\mathcal {L}(u(t)) = P^{(2m)}_2$$ for any $$t \in \mathbb {R}$$. $$\square $$

Finally, we show that the random variable *u* is indeed a global-in-time distributional solution to the Wick ordered NLS

5.15 $$\begin{aligned} i \partial _tu + \Delta u = \, :\!|u|^{2(m-1)} u\!: \;, \qquad (t,x) \in \mathbb {R}\times \mathcal {M}. \end{aligned}$$

#### Lemma 5.9

Let $\widetilde{u^{N_j}}$ and *u* be as above. Then, $\widetilde{u^{N_j}}$ and *u* are global-in-time distributional solutions to the truncated Wick ordered NLS (5.1) for each $j \in \mathbb {N}$ and to the Wick ordered NLS (5.15), respectively, almost surely with respect to $\widetilde{P}$.

#### Proof

For $j \in \mathbb {N}$, define the $\mathcal D'_{t, x}$-valued random variable $X_j$ by

$$\begin{aligned} X_j = i \partial _tu^{N_j} + \Delta u^{N_j} - \mathbf {P}_{N_j}\big (\! :\!|\mathbf {P}_{N_j}u^{N_j}|^{2(m-1)} \mathbf {P}_{N_j}u^{N_j}\!: \!\big ). \end{aligned}$$

Here, $\mathcal D'_{t, x}= \mathcal {D}'(\mathbb {R}\times \mathcal {M})$ denotes the space of space-time distributions on $\mathbb {R}\times \mathcal {M}$. We define $\widetilde{X}_j$ for $\widetilde{u^{N_j}}$ in an analogous manner. Since $u^{N_j}$ is a solution to (5.1), we see that $\mathcal {L}_{\mathcal D'_{t, x}}(X_j) = \delta _0$, where $\delta _0$ denotes the Dirac delta measure. By (5.11), we also have

$$\begin{aligned} \mathcal {L}_{\mathcal D'_{t, x}}(\widetilde{X}_j) = \delta _0, \end{aligned}$$

for each $j \in \mathbb {N}$. In particular, $\widetilde{u^{N_j}}$ is a global-in-time distributional solution to the truncated Wick ordered NLS (5.1) for each $j \in \mathbb {N}$, i.e.

$$\begin{aligned} i \partial _t\widetilde{u^{N_j}} + \Delta \widetilde{u^{N_j}} = \mathbf {P}_{N_j}\big (\! :\!|\mathbf {P}_{N_j} \widetilde{u^{N_j}}|^{2(m-1)} \mathbf {P}_{N_j}\widetilde{u^{N_j}}\!: \!\big ) \end{aligned}$$

in the distributional sense, almost surely with respect to $\widetilde{P}$.

In view of the almost sure convergence of $\widetilde{u^{N_j}}$ to *u* in $C(\mathbb {R}; H^s)$, we have

$$\begin{aligned} i \partial _t\widetilde{u^{N_j}} + \Delta \widetilde{u^{N_j}} \ \longrightarrow \ i \partial _tu + \Delta u \end{aligned}$$

in $\mathcal D'(\mathbb {R}\times \mathcal {M})$ as $j \rightarrow \infty$, almost surely with respect to $\widetilde{P}$. Next, we show the almost sure convergence of $F_{N_j}\big (\widetilde{u^{N_j}}\big )$ to $F(u) = \ :\!|u|^{2(m-1)} u\!:$. For simplicity of notation, let $F_j = F_{N_j}$ and $u_j = \widetilde{u^{N_j}}$. Given $M \in \mathbb {N}$, write

5.16 $$\begin{aligned} F_j (u_j) - F(u) =\,&\big ( F_j (u_j) - F(u_j) \big ) + \big ( F (u_j) - F_M(u_j) \big )\nonumber \\&+ \big ( F_M (u_j) - F_M(u) \big ) + \big ( F_M (u) - F(u) \big ). \end{aligned}$$

Then, for each fixed $M \ge 1$, it follows from the almost sure convergence of $\widetilde{u^{N_j}}$ to *u* in $C(\mathbb {R}; H^s)$ and the continuity of $F_M$ that the third term on the right-hand side of (5.16) converges to 0 in $C(\mathbb {R}; H^s)$ as $j \rightarrow \infty$, almost surely with respect to $\widetilde{P}$.

Fix $T>0$ and let $s < -1$. Arguing as in (5.7) with Proposition 4.6, we have

5.17 $$\begin{aligned} \big \Vert \Vert F (u_j) - F_M(u_j)\Vert _{L^2_T H^s}\big \Vert _{L^2(\nu _{N_j})}&= \big \Vert \Vert F (\Phi _{N_j} \phi ) - F_M(\Phi _{N_j} \phi )\Vert _{L^2(P^{(2m)}_{2, N_j}) H^s}\big \Vert _{L^2_T} \nonumber \\&= (2T)^\frac{1}{2} \Vert F ( \phi ) - F_M( \phi )\Vert _{L^2(P^{(2m)}_{2, N_j}) H^s} \nonumber \\&\lesssim T^\frac{1}{2} \Vert R_{N_j}\Vert _{L^4(\mu )} \Vert F ( \phi ) - F_M( \phi )\Vert _{L^4(\mu ) H^s} \nonumber \\&\le CT^{\frac{1}{2}} M^{-\varepsilon }, \end{aligned}$$

for some small $\varepsilon > 0$, uniformly in $j \in \mathbb {N}$. In the third step, we used the fact that $Z_N > rsim 1$ in view of Proposition 1.2: $Z_N = \Vert R_N(u) \Vert _{L^1(\rho )}\ \rightarrow \Vert R(u) \Vert _{L^1(\rho )}>0$ as $N \rightarrow \infty$. The fourth term on the right-hand side of (5.16) can be treated in an analogous manner. Proceeding as in (5.17), we obtain

$$\begin{aligned} \big \Vert \Vert F_j (u_j) - F(u_j)\Vert _{L^2_T H^s}\big \Vert _{L^2(\nu _{N_j})}&\le (2T)^\frac{1}{2} \Vert R_{N_j}\Vert _{L^4(\mu )} \Vert F_j ( \phi ) - F( \phi )\Vert _{L^4(\mu ) H^s} \nonumber \\&\le CT^{\frac{1}{2}} N_j^{-\varepsilon }. \end{aligned}$$

Putting everything together, we conclude that, after passing to a subsequence, $$F_j(u_j)$$ converges to *F*(*u*) in $$L^2([-T, T]; H^s)$$ almost surely with respect to $$\widetilde{P}$$. Since the choice of $$T>0$$ was arbitrary, we can apply the previous argument iteratively for $$T_\ell = 2^\ell $$, $$\ell \in \mathbb {N}$$. Thus, for each $$\ell \ge 2$$, we obtain a set $$\Omega _{\ell } \subset \Omega _{\ell -1} $$ of full measure such that a subsequence $$F_{j^{(\ell )}}(u_{j^{(\ell )}})(\omega ) $$ of $$F_{j^{(\ell -1)}}(u_{j^{(\ell -1)}}) $$ from the previous step converges to $$F(u)(\omega )$$ in $$L^2([-T_\ell , T_\ell ]; H^s)$$ for all $$\omega \in \Omega _\ell $$. Then, by a diagonal argument, passing to a subsequence, $$F_j(u_j) $$ converges to *F*(*u*) in $$L^2_\text {loc} H^s$$ almost surely with respect to $$\widetilde{P}$$. In particular, up to a subsequence, $$F_j(u_j) $$ converges to *F*(*u*) in $$\mathcal D'(\mathbb {R}\times \mathcal {M})$$ almost surely with respect to $$\widetilde{P}$$. Therefore, *u* is a global-in-time distributional solution to (5.15) almost surely with respect to $$\widetilde{P}$$. $$\square $$

Lemma 5.9 in particular implies that there exists $\Omega ' \subset \widetilde{\Omega }$ with $\widetilde{P}(\Omega ') = 1$ such that *u* is a global-in-time distributional solution to (5.15) for all $\omega \in \Omega '$. We now define the set $\Sigma$ in Theorem 1.5 as follows. With $\phi ={u}(0)$, we define $\Sigma =\phi (\Omega ') = R_0\circ u (\Omega ')$, where $R_0$ is the evaluation map at time $t = 0$ defined in Lemma 5.8 and *u* is viewed as a map$:\omega \in \widetilde{\Omega } \mapsto u \in C(\mathbb {R}; H^s)$. Then, by Lemma 5.8, we have

$$\begin{aligned} P^{(2m)}_2(\Sigma ) = (\widetilde{P} \circ u^{-1}) \circ R_0^{-1} (\Sigma ) = \widetilde{P}(\Omega ') = 1.\end{aligned}$$

With this definition of $\Sigma$, Theorem 1.5 follows from Lemmas 5.8 and 5.9.

## Appendix A: Example of a concrete combinatorial argument: the case $\mathcal {M}=\mathbb {T}^2$ and $m=3$

In this appendix, we present a concrete combinatorial computation on the Fourier side for the proof of Proposition 1.1 when $m= 3$. The aim of this appendix is to convince readers of increasing combinatorial complexity in *m* . Compare the $m=3$ case presented here with the $m=2$ case in [7]. This shows that the use of the white noise functional is essential in establishing our result for general $m \ge 2$.

Let $G_N(u)$ be as in (1.26). For simplicity, we show that $G_N(u)$ is uniformly bounded in $L^2(\mu )$. Namely, we prove

A.1 $$\begin{aligned} \Vert G_N(u) \Vert _{L^2(\mu )} \le C < \infty \end{aligned}$$

independently of $N \in \mathbb {N}$. Then, a small modification yields Proposition 1.1 for $p = 2$. The general case follows from the $p = 2$ case and the Wiener chaos estimate (Lemma 2.6).

From (1.21), (1.23), (1.24), and (1.26) with (1.10), we have

A.2 $$\begin{aligned} 6 G_N(u)&= \int _{\mathbb {T}^2} :\! |u_N|^6 \!: dx = \int _{\mathbb {T}^2} |u_N|^6 - 9 \sigma _N |u_N|^4 +18 \sigma _N^2 |u_N|^2 - 6 \sigma _N^3 dx \nonumber \\&= \sum _{\begin{array}{c} \Gamma _{6}(0)\\ |n_j| \le N \end{array}}\prod _{j = 1}^{6} \frac{g^*_{n_j}}{\sqrt{1+|n_j|^2}} - 9 \left( \sum _{|n|\le N}\frac{1}{1+|n|^2} \right) \left( \sum _{\begin{array}{c} \Gamma _{4}(0)\\ |n_j| \le N \end{array}}\prod _{j = 1}^{4} \frac{g^*_{n_j}}{\sqrt{1+|n_j|^2}}\right) \nonumber \\&\quad +18 \left( \sum _{|n|\le N}\frac{1}{1+|n|^2} \right) ^2 \left( \sum _{|n|\le N}\frac{|g_n|^2}{1+|n|^2} \right) -6 \left( \sum _{|n|\le N}\frac{1}{1+|n|^2} \right) ^3\nonumber \\&=: \text {I}+ \text {II} + \text {III}+ \text {IV}, \end{aligned}$$

where $\sigma _N$ is as in (1.25) and $\Gamma _k(0)$ and $g_n^*$ are as in (2.29) and (2.30), respectively.

The basic idea is to regroup the terms in (A.2) by introducing some factorizations, and separately estimate each contribution. Given $\ell \in 2\mathbb {N}$, we say that we have a *pair* in $\overline{n} = (n_1, \dots , n_\ell ) \in \Gamma _{\ell } (0)$ if $n_j = n_{j'}$ for some odd *j* and even $j'$.

Let us first consider $\text {I}$. Given $\overline{n} \in \Gamma _6(0)$, there are three cases: (i) no pair, (ii) 1 pair, and (iii) 3 pairs. Thus, write $\text {I}$ as

$$\begin{aligned} \text {I}= \text {I}_1 + \text {I}_2 + \text {I}_3, \end{aligned}$$

corresponding to the three cases: (i) no pair, (ii) 1 pair, and (iii) 3 pairs, respectively. For simplicity of notation, we may drop the frequency restriction $|n| \le N$ in the following but it is understood that all the summations are over $\{|n|\le N\}$.

$\bullet$ *Case 1* No pair.    In this case, we can easily estimate the contribution from $\text {I}_1$ by

A.3 $$\begin{aligned} \Vert \text {I}_1\Vert _{L^2(\mu )} \lesssim \left( \sum _{\Gamma _{6}(0)}\prod _{j = 1}^{6} \frac{1}{1+|n_j|^2}\right) ^\frac{1}{2} \le C < \infty . \end{aligned}$$

$\bullet$ *Case 2* 1 pair.    In this case, there are 9 possibilities to form a pair from each of $\{n_1, n_3, n_5\}$ and $\{n_2, n_4, n_6\}$. Thus, we have

$$\begin{aligned} \text {I}_2 = 9 \left( \sum \frac{|g_n|^2}{1+|n|^2} \right) \left( \sum _{\begin{array}{c} \Gamma _{4}(0)\\ n_1 \ne n_2, n_4 \end{array}}\prod _{j = 1}^{4} \frac{g^*_{n_j}}{\sqrt{1+|n_j|^2}}\right) . \end{aligned}$$

Combining this with $\text {II}$, we have

A.4 $$\begin{aligned} \text {I}_2 +\text {II}&= 9 \left( \sum \frac{|g_n|^2 - 1}{1+|n|^2} \right) \left( \sum _{\begin{array}{c} \Gamma _{4}(0)\\ n_1 \ne n_2, n_4 \end{array}}\prod _{j = 1}^{4} \frac{g^*_{n_j}}{\sqrt{1+|n_j|^2}}\right) \nonumber \\&\quad - 18 \left( \sum \frac{1}{1+|n|^2} \right) \left( \sum \frac{|g_n|^2}{1+|n|^2} \right) ^2\nonumber \\&\quad +9 \left( \sum \frac{1}{1+|n|^2} \right) \left( \sum \frac{|g_n|^4}{(1+|n|^2)^2} \right) \nonumber \\&=: \text {II} _1+\text {II} _2 + \text {II} _3. \end{aligned}$$

Note that $\mathbb {E}[|g_n|^2 - 1] = 0$. Then, by Lemma 2.6, we have

A.5 $$\begin{aligned} \Vert \text {II} _1\Vert _{L^2(\mu ) }&\lesssim \bigg \Vert \sum \frac{|g_n|^2 - 1}{1+|n|^2} \bigg \Vert _{L^4(\mu )} \bigg \Vert \sum _{\begin{array}{c} \Gamma _{4}(0)\\ n_1 \ne n_2, n_4 \end{array}}\prod _{j = 1}^{4} \frac{g^*_{n_j}}{\sqrt{1+|n_j|^2}} \bigg \Vert _{L^4(\mu )} \nonumber \\&\lesssim \bigg ( \sum \frac{1}{(1+|n|^2)^2} \bigg )^\frac{1}{2} \bigg ( \sum _{\Gamma _{4}(0)}\prod _{j = 1}^{4} \frac{1}{1+|n_j|^2}\bigg )^\frac{1}{2} \le C < \infty . \end{aligned}$$

The terms $\text {II} _2$ and $\text {II} _3$ are treated with other terms in the following.

$\bullet$ *Case 3* 3 pairs.    In this case, there are 3 scenarios on the values of $n_1, n_3$, and $n_5$: (i) $n_1 = n_3 = n_5$, (ii) $n_1 = n_3\ne n_5$ up to permutations, (iii) all distinct. Write $\text {I}_3 = \text {I}_{31} + \text {I}_{32} + \text {I}_{33}$, corresponding to these three cases.

$\circ$ Subcase 3 (i): $n_1 = n_3 = n_5$.    In this case, the contribution can be estimated by

A.6 $$\begin{aligned} \Vert \text {I}_{31}\Vert _{L^2(\mu ) }&\le \bigg \Vert \sum \frac{|g_n|^6 }{(1+|n|^2)^3} \bigg \Vert _{L^2(\mu )} \lesssim \bigg ( \sum \frac{1 }{(1+|n|^2)^6} \bigg )^\frac{1}{2} \le C < \infty . \end{aligned}$$

$\circ$ Subcase 3 (ii): $n_1 = n_3\ne n_5$ up to permutations.    In this case, we have

A.7 $$\begin{aligned} \text {I}_{32}&= \begin{pmatrix}3\\ 2\end{pmatrix} \begin{pmatrix}3\\ 2\end{pmatrix} \bigg ( \sum \frac{|g_n|^4 }{(1+|n|^2)^2} \bigg ) \bigg ( \sum _{m \ne n} \frac{|g_m|^2 }{1+|m|^2} \bigg ) \nonumber \\&= 9 \, \bigg ( \sum \frac{|g_n|^4 }{(1+|n|^2)^2} \bigg ) \bigg ( \sum \frac{|g_m|^2 }{1+|m|^2} \bigg ) -9 \bigg ( \sum \frac{|g_n|^6 }{(1+|n|^2)^3} \bigg )\nonumber \\&=: \text {I}_{321} + O_{L^2(\mu ) }(1). \end{aligned}$$

Here, we estimated the second term as in (A.6).

$\circ$ Subcase 3 (iii): all distinct.    In this case, we have

A.8 $$\begin{aligned} \text {I}_{33}&= 6\, \bigg ( \sum \frac{|g_{n_1}|^2 }{1+|n_1|^2} \bigg ) \bigg ( \sum _{n_3 \ne n_1} \frac{|g_{n_3}|^2 }{1+|n_3|^2} \bigg ) \bigg ( \sum _{n_5 \ne n_1, n_3} \frac{|g_{n_5}|^2 }{1+|n_5|^2} \bigg )\nonumber \\&= 6\, \bigg ( \sum \frac{|g_{n_1}|^2 }{1+|n_1|^2} \bigg ) \bigg ( \sum _{n_3 \ne n_1} \frac{|g_{n_3}|^2 }{1+|n_3|^2} \bigg ) \bigg ( \sum _{n_5 \ne n_1} \frac{|g_{n_5}|^2 }{1+|n_5|^2} \bigg )\nonumber \\&\quad -\,6 \, \bigg ( \sum \frac{|g_{n_1}|^2 }{1+|n_1|^2} \bigg ) \bigg ( \sum _{n_3 \ne n_1} \frac{|g_{n_3}|^4 }{(1+|n_3|^2)^2} \bigg ) \nonumber \\&= 6\, \bigg ( \sum \frac{|g_{n_1}|^2 }{1+|n_1|^2} \bigg ) \bigg ( \sum _{n_3 \ne n_1} \frac{|g_{n_3}|^2 }{1+|n_3|^2} \bigg ) \bigg ( \sum \frac{|g_{n_5}|^2 }{1+|n_5|^2} \bigg )\nonumber \\&\quad -\,6\, \bigg ( \sum \frac{|g_{n_1}|^2 }{1+|n_1|^2} \bigg ) \bigg ( \sum _{n_3 \ne n_1} \frac{|g_{n_3}|^4 }{(1+|n_3|^2)^2} \bigg ) \nonumber \\&\quad -\,6 \, \bigg ( \sum \frac{|g_{n_1}|^4 }{(1+|n_1|^2)^2} \bigg ) \bigg ( \sum _{n_3 \ne n_1} \frac{|g_{n_3}|^2 }{1+|n_3|^2} \bigg ) \nonumber \\&= 6\, \bigg ( \sum \frac{|g_{n_1}|^2 }{1+|n_1|^2} \bigg )^3 -18 \bigg ( \sum \frac{|g_{n_1}|^2 }{1+|n_1|^2} \bigg ) \bigg ( \sum \frac{|g_{n_3}|^4 }{(1+|n_3|^2)^2} \bigg ) \nonumber \\&\quad +\,12\, \bigg ( \sum \frac{|g_{n_1}|^6 }{(1+|n_1|^2)^3} \bigg )\nonumber \\&=: \text {I}_{331}+\text {I}_{332} + O_{L^2(\mu ) }(1). \end{aligned}$$

From (A.4), (A.7), and (A.8), we have

$$\begin{aligned} \text {II} _3 + \text {I}_{321}+ \text {I}_{332} = 9 \bigg ( \sum \frac{1-|g_n|^2}{1+|n|^2} \bigg ) \bigg ( \sum \frac{|g_n|^4}{(1+|n|^2)^2} \bigg ). \end{aligned}$$

Proceeding as in (A.5), we obtain

A.9 $$\begin{aligned} \Vert \text {II} _3 + \text {I}_{321}+ \text {I}_{332}\Vert _{L^2(\mu ) }\le C < \infty . \end{aligned}$$

From (A.2), (A.4), and (A.8), we have

$$\begin{aligned} \text {III}+ \text {IV}+ \text {II} _2 + \text {I}_{331} = 6\, \bigg ( \sum \frac{|g_n|^2-1}{1+|n|^2} \bigg )^3. \end{aligned}$$

Proceeding as in (A.5), we obtain

A.10 $$\begin{aligned} \Vert \text {III}+ \text {IV}+ \text {II} _2 + \text {I}_{331} \Vert _{L^2(\mu ) }&\lesssim \bigg \Vert \sum \frac{|g_n|^2 - 1}{1+|n|^2} \bigg \Vert _{L^6(\mu )}^3 \le C < \infty . \end{aligned}$$

Finally, putting (A.2)–(A.10) together, we obtain (A.1).

### Remark A.1

The above computation merely handles the nonlinear part $G_N(u)$ in the truncated Wick ordered Hamiltonian. In order to prove Theorem 1.4, one still needs to estimate $F_N(u)$ in (1.36), which has a different combinatorial structure. For our problem, it is much more efficient to work on the physical side, using the white noise functional and the (generalized) Laguerre polynomials.

## Footnotes

1 With the exception of the Wiener chaos estimate (Lemma 2.6).

2 In the following, *Z*, $Z_N$, and etc. denote various normalizing constants so that the corresponding measures are probability measures when appropriate.

3 For simplicity, we set $\beta = 1$ in the following. See [34] for a discussion on the Gibbs measures and different values of $\beta > 0$.

4 Strictly speaking, there is a factor of $(2\pi )^{-1}$ in (1.10). For simplicity of the presentation, however, we drop such harmless $2\pi$ hereafter.

5 Namely, $g_n$ has mean 0 and $\text {Var}(g_n) = 1$.

6 In the case of NLW, we only need to use the Hermite polynomials since we deal with real-valued functions.

7 Indeed, the discussion presented here also holds for $d = \infty$ in the context of abstract Wiener spaces. For simplicity, however, we restrict our attention to finite values for *d*.

8 Here, $Q_\mathcal {H}= \mathbb {R}^d$ when $d < \infty$. When $d = \infty$, we set $Q_\mathcal {H}$ to be an appropriate extension of $\mathcal {H}$ such that $(\mathcal {H}, Q_\mathcal {H}, \mu _\infty )$ forms an abstract Wiener space with $\mathcal {H}$ as the Cameron-Martin space.

9 This is (equivalent to) the Fock space in quantum field theory. See [39, Chapter I]. In particular, the Fock space $\mathcal {F}(\mathcal {H}) = \bigoplus _{k = 0}^\infty \mathcal {H}_\mathbb {C}^{\otimes _\text {sym}^k}$ is shown to be equivalent to the Wiener–Ito decomposition (2.5). In the Fock space formalism, the Wick renormalization can be stated as the reordering of the creation operators on the left and annihilation operator on the right. We point out that while much of our discussion can be recast in the Fock space formalism, our main aim of this paper is to give a self-contained presentation (as much as possible) accessible to readers not familiar with the formalism in quantum field theory. Therefore, we stick to a simpler Fourier analytic and probabilistic approach.
